# Co-infection by Soil-Borne Fungal Pathogens Alters Disease Responses Among Diverse Alfalfa Varieties

**DOI:** 10.3389/fmicb.2021.664385

**Published:** 2021-07-14

**Authors:** Xiangling Fang, Caixia Zhang, Zi Wang, Tingyu Duan, Binhua Yu, Xitao Jia, Jiayin Pang, Lisong Ma, Yanrong Wang, Zhibiao Nan

**Affiliations:** ^1^State Key Laboratory of Grassland Agro-ecosystems, Key Laboratory of Grassland Livestock Industry Innovation, Ministry of Agriculture and Rural Affairs, College of Pastoral Agriculture Science and Technology, Lanzhou University, Lanzhou, China; ^2^School of Agriculture and Environment, UWA Institute of Agriculture, The University of Western Australia, Perth, WA, Australia; ^3^Division of Plant Science, Research School of Biology, Australian National University, Canberra, ACT, Australia

**Keywords:** *Fusarium oxysporum*, *Rhizoctonia solani*, co-infection, single infection, wilt, root rot, alfalfa, host disease resistance

## Abstract

*Fusarium oxysporum* f. sp. *medicaginis* (Fom) and *Rhizoctonia solani* (Rs) are the major soil-borne fungal pathogens that pose severe threats to commercial alfalfa production in China. However, the effects of Fom and Rs co-infection on alfalfa and whether co-infection alters disease resistance responses among diverse varieties remain unknown. A collection of 80 alfalfa varieties (*Medicago sativa*) originated from seven countries were used to study the effects of Fom and Rs co-infection on alfalfa and host resistance responses. The co-infection resulted in more severe disease and reductions in growth and biomass allocation across varieties in comparison with either single infection by Fom or Rs; in addition, root morphology was much more strongly altered by the co-infection. Principal component analysis based on all plant traits showed that varieties under the co-infection were related to the single infection by Rs, being separated from Fom, and hierarchical clustering found differential response patterns among varieties upon co-infection compared with either single infection, with most varieties being highly susceptible to the co-infection. Furthermore, varieties that were most resistant to either single infection were not effective to co-infection, and there was no individual variety with resistance to both pathogens singly and co-infected. This study reveals for the first time that the co-infection by Fom and Rs alters disease resistance responses among diverse alfalfa varieties and provides useful information for developing alfalfa varieties with resistance to the co-occurrence of different soil-borne pathogens.

## Introduction

Alfalfa (*Medicago sativa*) is the most widely grown forage crop worldwide with great economic and ecological importance (Yang et al., 2008). It is a perennial herbaceous legume that has been cultivated for more than 2,000 years in over 80 countries with a global production area over 32 million hectares (Chen et al., 2020). Besides its widely acknowledged characters such as “King of Forages” with high yield, rich nutrition, and wide adaptability, it plays an essential role in the ecosystem by conserving soil and water as well as improving soil structure and fertility (Yang et al., 2008; Fang et al., 2019; Chen et al., 2020). It also has the potential to improve global food security (Khoury et al., 2014). In China, alfalfa ranks first in cultivated grassland, and the demand for alfalfa forage over the last 50 years has largely increased, with the production area reaching 4 million hectares (Bai et al., 2018; Fang et al., 2019). It makes important contributions to the development of grassland animal husbandry and the sustainability of agricultural ecosystems in China (Fang et al., 2019). However, diseases such as wilt and root rot caused by soil-borne pathogens pose a major constraint to alfalfa production worldwide and in China. It causes an estimated annual yield loss of 20–40% in the world (Fang et al., 2019; Wang et al., 2020b). Wilt and root rot is prevalent in alfalfa fields established for more than 2 years and has a disease incidence ranging from 30 to 80% in northwest China (Fang et al., 2019; Wang et al., 2020b). So far, there are no effective control methods for soil-borne fungal pathogens in alfalfa.

*Fusarium oxysporum* f. sp. medicaginis (Fom) and *Rhizoctonia solani* (Rs) are the most frequently occurring and damaging soil-borne pathogens that are major concerns in alfalfa in China (Li et al., 2009; Huang et al., 2013; Guo et al., 2015; Xin et al., 2016; Fang et al., 2019; Luo et al., 2020). Worldwide, *F. oxysporum* (Fo) and Rs are also the major pathogens in a wide range of economically important crops such as grain legumes and forage legumes (Fravel et al., 2003; Michielse and Rep, 2009; Hane et al., 2014; Ajayi-Oyetunde and Bradley, 2018; Wille et al., 2019). Both pathogens penetrate plants through roots and can infect plants at any growth stage; besides, they are necrotrophic/saprophytic pathogens that persist in soil and plant residues for long periods in the absence of plant hosts (Berrocal-Lobo and Molina, 2008; Michielse and Rep, 2009; Drizou et al., 2017; Gordon, 2017; Ajayi-Oyetunde and Bradley, 2018; Wille et al., 2019). The genetic complexity of Fo and Rs further hinders the plant breeding from developing resistant cultivars (Berrocal-Lobo and Molina, 2008; Anderson et al., 2013; Ajayi-Oyetunde and Bradley, 2018). Fo, mainly associated with wilt disease, ranks among the top 10 most economically important fungal pathogens and has been divided into more than 100 *formae speciales* (f. spp.) based on its host specificity (Michielse and Rep, 2009; Dean et al., 2012; Edel-Hermann and Lecomte, 2019). Fom is the host-specific pathogen of alfalfa and other *Medicago* spp. (Rispail and Rubiales, 2014; Gill et al., 2018; Batnini et al., 2020). Rs, mainly associated with root rot disease, has been classified into 14 anastomosis groups based on hyphal anastomosis reactions (Ajayi-Oyetunde and Bradley, 2018; Li et al., 2019). Extensive studies have been performed on Fo and Rs in many crops, and studies are often limited to a single pathogen with its host (Gordon, 2017; Ajayi-Oyetunde and Bradley, 2018; Fang et al., 2019; Oladzad et al., 2019; Batnini et al., 2020). However, the combined effects of both pathogens on plants and how plants respond to the co-infection remain unknown.

Soil-borne pathogens are difficult to control due to the complexity and longevity of the causal pathogens that can survive for many years either in soils or on plant residues (Fang and Barbetti, 2014; Ajayi-Oyetunde and Bradley, 2018; Wille et al., 2019). It is much more difficult in alfalfa, which is a perennial crop as the pathogens also survive and accumulate within the root tissue over the years (Fang et al., 2019). The major control methods like chemical control are not feasible or economic for soil-borne diseases in most crops (Haas and Défago, 2005; Hane et al., 2014; Fang et al., 2019). Breeding crop varieties with effective disease resistance has been considered as the most efficient, economic, and environmentally sustainable approach to manage to soil-borne diseases (Rubiales et al., 2015; Gordon, 2017; Fang et al., 2019; Wille et al., 2019). Although substantial progress has been made in developing varieties with resistance to a single pathogen in many other crop species, it has been indicated that these resistances are often overcome in the field where usually more than one pathogen is prevalent, especially for soil-borne pathogens (Tollenaere et al., 2016; Wille et al., 2019). It has been highlighted that plants simultaneously infected by several pathogens represent a widespread occurrence in agriculture (Tollenaere et al., 2016; Abdullah et al., 2017). However, developing varieties with resistance to multiple pathogens that reflects the field situations is far behind that to a single pathogen due to very limited knowledge of the co-infection effects on disease epidemics and plant growth in plant pathosystems (Tollenaere et al., 2016; Abdullah et al., 2017; Wille et al., 2019). Knowledge on the co-infection by different pathogens will aid the way in developing more sustainable solutions to reduce threats caused by soil-borne pathogens and improving disease management (Wille et al., 2019).

Therefore, there is an urgent need to study crop diseases caused by the co-infection of different pathogens, apart from always focusing on a single pathogen (Tollenaere et al., 2016; Abdullah et al., 2017; Wille et al., 2019). Different pathogen species can infect plants simultaneously and likely tends to different disease epidemics in comparison with the infection by a single pathogen (Susi et al., 2015; Abdullah et al., 2017; Wille et al., 2019). Previous studies showed that plants co-infected by different soil-borne pathogens displayed a significant increase in disease development compared with single infection (Kerr, 1963; Pfender and Hagedorn, 1982; Peters and Grau, 2002). It has also been reported that co-infection by different soil-borne pathogens did not result in more severe disease than single infection (Vandemark et al., 2010; Lerch-Olson and Robertson, 2020). However, previous studies on the co-infection effects by different soil-borne pathogens on disease epidemics involve only single or several varieties (Wille et al., 2019). So far, it is unknown whether the co-infection by soil-borne pathogens alters disease resistance response among diverse varieties of a crop species. In addition, comprehensive evaluations of plant resistance responses to soil-borne pathogens based on multiple traits of both disease severity and growth performance rather than single disease traits are urgently needed. Membership function method has been widely used for accurately evaluating plant tolerance responses among diverse varieties to abiotic stresses based on multiple traits and has been applied to many crops such as wheat and soybean (Liu et al., 2017; Song et al., 2017; Yan et al., 2020).

To date, the effects of Fom and Rs co-infection on alfalfa and whether co-infection alters disease resistance responses among diverse varieties remains unknown. The membership function method has not been used much in the evaluation of plant resistance responses to biotic stresses such as soil-borne fungal pathogens. This study, for the first time, determined the effects of Fom and Rs co-infection on a collection of 80 cultivated alfalfa varieties based on plant traits associated with disease severity, plant growth and biomass allocation, and evaluated how these varieties responded to Fom and Rs under co-infection in comparison with either single infection based on the membership function method.

## Materials and Methods

### Alfalfa Varieties

A collection of 80 alfalfa varieties (*M. sativa*) with 75 belonging to *M. sativa* subsp. *sativa* and 5 belonging to *M. sativa* subsp. *varia* were used in this study (Supplementary Table 1). Varieties of *M. sativa* subsp. *sativa* includes 24 domestic bred and 4 local varieties in China, and 47 introduced varieties from six countries (Austria, Australia, Canada, France, Guatemala, and United States), and varieties of *M. sativa* subsp. *varia* are all domestic bred varieties. All the domestic and local varieties except four new ones were commercially released varieties in China during the last 30 years. The local varieties, e.g., Longdong, have been cultivated in Loess Plateau areas in China for more than 2,000 years (Sun et al., 2016). Most varieties have agronomic characteristics such as tolerance to abiotic stresses (drought, cold, and/or salinity) and/or good quality, and are suitable for cultivation in different geographic areas in China (Supplementary Table 1).

### Fungal Isolates, Identification, and Inoculum Preparation

One single-spored isolate of *F. oxysporum* f. sp. *medicaginis* (Fom) and one single-hyphal-tip isolate of *R. solani* (Rs) were used in this study. The isolates were deposited in the Grassland Culture Collection Centre, Lanzhou University, China, with accession nos. LZU-MsR-Fo.LZ22 and LZU-MsR-Rs.YLQ5, respectively. The two isolates were representative isolates of Fom and Rs recovered from root tissues of moderately stunted plants collected from commercial alfalfa fields in 2017 in Gansu province, China. The Fom isolate was identified based on cultural and conidial morphology as well as molecular sequencing of the internal transcribed spacer region (ITS) and translation elongation factor 1 alpha (EF1 alpha) (O’Donnell et al., 2009; Vu et al., 2019). The Rs isolate was identified based on cultural and hyphal morphology and then molecular sequencing of ITS (Fang et al., 2013). The ITS and EF1 alpha sequences of Fom and the ITS sequence of Rs were deposited in GenBank with accession nos. MW036290, MW560899, and MW036291, respectively. In addition, both isolates were previously confirmed as pathogens to alfalfa as they caused disease on the local cultivated varieties such as Longdong and Longzhong grown in Gansu province.

The Fom isolate was preserved as mycelia-colonized pieces of filter paper dried at room temperature and stored at −20°C (Fang and Barbetti, 2014). The Rs isolate was preserved as mycelial-lyophilized cultures on colonized millet seeds in glass ampoules at 4°C (Fang et al., 2013). Isolates were sub-cultured onto fresh 1/5 strength potato dextrose agar (PDA) plates when needed for inoculum preparation. Millet seed-based inoculum of each isolate was prepared according to the modified procedure as described (Fang et al., 2013). In brief, 50 g of millet seeds (*Panicum miliaceum*) was soaked in deionized water (DI) water in a 250-ml flask for 10 h (excess water was drained) and then autoclaved at 121°C for 20 min on three consecutive days. Six 3-mm-diameter agar pieces from margins of 5-day-old colonies of Fom and Rs growing on PDA plates were sub-cultured into flasks containing autoclaved millet seeds. Flasks were incubated at 22°C in darkness for 2 weeks and were shaken every 2 days to ensure uniform colonization.

### Pathogen Treatments and Growth Conditions

The experiment consisted of three pathogen treatments (viz., two single inoculations and one co-inoculation) and one control treatment for comparison without pathogen. For the single-pathogen treatment, the soil was inoculated with millet seed-based inoculum of Fom or Rs at a rate of 0.5% (w/w); for the co-inoculation treatment, the soil was inoculated with the mixture of both isolates at 1:1 ratio (Fom:Rs). Under each treatment, there were four pot replicates, and each pot contained four seedlings for each variety. The soil used in this study was a soil mixture of the loessal soil and commercial peat soil (Pindstrup Mosebrug A/S, Denmark) at 3:1 ratio (w/w). The loessal soil, the dominant soil type in the cropping area in Loess Plateau, China (Cao et al., 2011), was collected from the top 20 cm in a local alfalfa field, Yuzhong County, China. The field soil was air-dried and then passed through a 2-mm mesh sieve to remove debris before being mixed thoroughly with peat soil. The soil mixture was then autoclaved at 121°C for 20 min on three consecutive days before use. Seeds of each variety were surface sterilized in 70% ethanol for 30 s, washed at least three times with sterilized DI water, and then sown in seedling trays with autoclaved soil mixture at a depth of 0.5 cm. Two-week-old alfalfa seedlings at the three- to four-leaf stage were removed from trays and washed with sterilized DI water before being transplanted into pots (12 cm × 12 cm) filled with soil of each treatment. All pots were maintained under the same conditions and watered every 2 days to free draining with DI water. The bottom of each pot had four 5-mm-diameter holes to allow free drainage, which was covered by a nylon mesh to prevent any soil leakage. The experiment was conducted in controlled environment rooms at 25°C/15°C (day/night) with a 12-h photoperiod and 65% relative humidity. The experiment was arranged in a randomized block design and was repeated two times under the same conditions.

### Plant Measurements and Pathogen Re-isolation

One day prior to the final harvest at 1 month, shoot symptoms and plant heights were recorded for each treatment. Shoot symptoms were recorded based on a 0–5 scale as previously described (Fang et al., 2011; Fang and Barbetti, 2012), where 0 = plant well developed, no disease symptoms; 1 = plant slightly stunted; 2 = plant stunted and/or yellowing; 3 = plant severely stunted and/or wilting; 4 = majority of leaves wilted or dead; 5 = plant dead. Plant heights were measured from the base to top of the shoots. Whole plants were then removed from pots and thoroughly washed under running tap water to remove any attached soil. Plants were then floated in shallow trays with DI water to remove any remaining soil residues and blot-dried by towel paper. Root symptoms were scored according to a 0–5 severity scale (Fang and Barbetti, 2012; Fang et al., 2013), where 0 = root well developed, no discoloration and/or rot; 1 ≤25% root discolored; 2 = 25–50% root discolored and/or rotted; 3 = 50–75% root discolored and/or rotted; 4 = ≥75% root discolored/rotted; 5 = all root discolored/rotted off. Plants from each pot were separated into shoots and roots, placed in separate paper bags, and then dried at 69°C for 1 week before weighing.

Immediately after harvest and disease assessment, 12 varieties (Msv4, Msv16, Msv26, Msv21, Msv50, Msv53, Msv56, Msv61, Msv62, Msv69, Msv71, and Msv79) were randomly selected for the measurements of root morphological traits. The entire root system of each plant was spread out in DI water in a transparent plastic tray (30 × 20 × 2 cm) and scanned at 600 dpi using a flatbed scanner (Epson Perfection V850 Pro). Root images were analyzed for root diameter (RD), total root length (TRL), root surface area (RSA), and root volume (RV) using WinRHIZO Pro software (Version 2019a, Regent Instruments Inc.).

In addition, re-isolations of the pathogens were performed to confirm that disease symptoms were caused by the inoculated isolates. Root segments of the 12 randomly selected varieties were superficially sterilized with 1.25% sodium hypochlorite for 30 s and then rinsed three times in sterile DI water before culturing on PDA plates (Fang et al., 2011). The cultures were examined microscopically, which showed morphological identities with the inoculated isolates and then molecular identification by ITS sequencing. The sequence identities between the re-isolated and inoculated isolates of Fom and Rs were confirmed by direct pairwise comparisons (Molecular Evolutionary Genetics Analyses software, Version 10.0).

### Data Analyses and Membership Function

Under each pathogen treatment, disease severity index (DI%) of shoot and root for each variety was calculated based on the formula described (Fang et al., 2013): $D\,I\%=\left[\sum_{0}^{r}\left(T\,r\times r\right)/\left(T\times 5\right)\right]\times 100$, where *Tr* represents the number of plants for each disease rating, *r* represents the disease rating (*r* = 0, 1, 2, 3, 4, 5), and *T* represents the total number of plants. The number 5 in the denominator of the ratio corresponds to the maximum disease index value. Biomass allocation traits including root biomass ratio (RT) and shoot biomass ratio (ST) were calculated as the ratio of dry weight for root (DWR) and dry weight for shoot (DWS) to the dry weight for total plant, respectively. To define the intensity of pathogen effects on plant growth and biomass allocation, the effect size (ES) of each trait for each variety was calculated by the formula: *ES*_*t*.*v*._=(*X*_*t*.*v*.*ck*_−*X*_*t*.*v*.*p*_)/*X*_*t*.*v*.*ck*_×100, where *X*_*t.v.p*_ and *X*_*t.v.ck*_ are the values of the trait (*t*) for the variety (*v*) evaluated under each pathogen treatment (*p*) and the control treatment (*ck*), respectively. A positive value indicated that the trait was reduced in response to pathogen infection while a negative value indicated that the trait was increased in response to pathogen infection. All data on disease severity and effect sizes of growth and biomass allocation were log10-transformed before analyses of variance (ANOVAs), and the original (untransformed) data were presented. After transformation, data on each trait had a normal distribution and similar variation. Data from the two repeat experiments were combined and analyzed together since there were no significant differences (*p* = 0.12) in the data between the experiments. Data analyses were conducted using GenStat (version 15, VSN International Ltd., United Kingdom, 2019) and *R* (version 3.6.0, *R* Development Core Team, 2019).

Analyses of variance was conducted to examine the differences among pathogen treatment, variety treatment, and their interaction in disease severity, growth, and biomass allocation using the *R* package “*Agricolae*” (de Mendiburu, 2020). For disease severity, data under the control treatment (all 0) were not included in the analyses since the control plants showed no disease. Subsequent multiple comparisons among pathogen treatments were made using Fisher’s protected least significant differences (LSD) at *p* = 0.05. The differences in pathogen effect sizes of growth and biomass allocation traits among three pathogen treatments across varieties and among varieties under each pathogen treatment were also determined by ANOVA. Subsequent multiple comparisons among pathogen treatments were made using Fisher’s protected LSD at *p* = 0.05, with standard errors (SE) of means for each variety being computed.

To reveal possible coordination among different plant traits across varieties under each pathogen treatment, correlations among pathogen effect sizes of plant growth and biomass allocation traits with disease traits were determined by Pearson’s correlation analyses using the *R* Package “*Agricolae*” (de Mendiburu, 2020). Principal component analysis (PCA) on all plant traits for 80 varieties under three pathogen treatments compared with the control was constructed with the *R* package “*Factoextra*” (Kassambara and Mundt, 2020), and PCA on the pathogen effect sizes of plant traits for 80 varieties under each pathogen treatment was then performed. Hierarchical clustering was performed based on variations in pathogen effect size of plant traits under each pathogen treatment using the *R* Package “*Pheatmap*” (Kolde, 2019) with Bray–Curtis as the distance metric and Complete Linkage clustering as the linkage method.

The comprehensive resistance response of 80 alfalfa varieties to Fom and Rs under either single or co-infection was evaluated based on the membership function described with some modifications (Liu et al., 2017; Wang et al., 2020a). The modified membership function (Eq. 5) considers the weight of all plant traits associated with disease, growth, and biomass allocation for each variety. A larger *D*_*v*_ value indicates that the resistance of the variety is higher while a smaller value indicates that the resistance is lower. Varieties that were ranked in the top 10 based on *D*_*v*_ value were considered as the most resistant varieties under each pathogen treatment.

(1) $$D\,C_{t.v.}=X_{t.v.p}/X_{t.v.c\,k}$$

(2) $$C\,D\,C_{v}=\frac{1}{n}\,\,\sum_{t=1}^{n}D\,C_{t.v}$$

(3) $$C\,D\,I_{v}=\frac{1}{n}\,\,\sum_{t=1}^{n}C\,D\,C_{v}\times D\,C_{t.v}/\,\bar{D\,C_{t.v}}$$

(4) $$U_{t.v}=\frac{D\,C_{t.v}-D\,C_{t\,m\,i\,n}}{D\,C_{t\,m\,a\,x}-D\,C_{t\,m\,i\,n}}$$

(5) $$D_{v}=\sum_{t=1}^{n}\,\left[U_{t.v}\times \left(\left|r\,t\right|/\,\sum_{t=1}^{n}\left|r\,t\right|\right)\right]$$

where *DC*_*t.v.*_ is the disease resistance coefficient of the trait (*t*) for the variety (*v*), and *X*_*t.v.p*_ and *X*_*t.v.ck*_ are the values of the trait (*t*) for the variety (*v*) evaluated under the pathogen treatment (*p*) and control treatment (*ck*), respectively. *CDC*_*v*_ is the comprehensive disease resistance coefficient for the variety (*v*), and *n* is the number of all investigated traits. *CDI*_*v*_ is the comprehensive disease resistance index for the variety (*v*), and $\bar{D\,C_{t.v}}$ is the mean *DC*_*t.v*_ of all varieties under the pathogen treatment. *DC*_*t.v*_, *CDCv*, $\bar{D\,C_{t.v}}$, and *CDI*_*v*_ were calculated for each pathogen treatment. *U*_*t.v*_ is the membership function value for disease resistance of the trait (*t*) for the variety (*v*), and *DC*_*tmin*_ and *DC*_*tmax*_ are the respective min and max values for the disease resistance coefficient of the trait (*t*). *D*_*v*_ is the comprehensive membership function value for disease resistance of the variety (*v*), and *rt* is the correlation coefficient between the disease resistance coefficient of the trait (*t*) and comprehensive disease resistance index of the variety (*v*).

## Results

### Disease Severity

**FIGURE 1:**
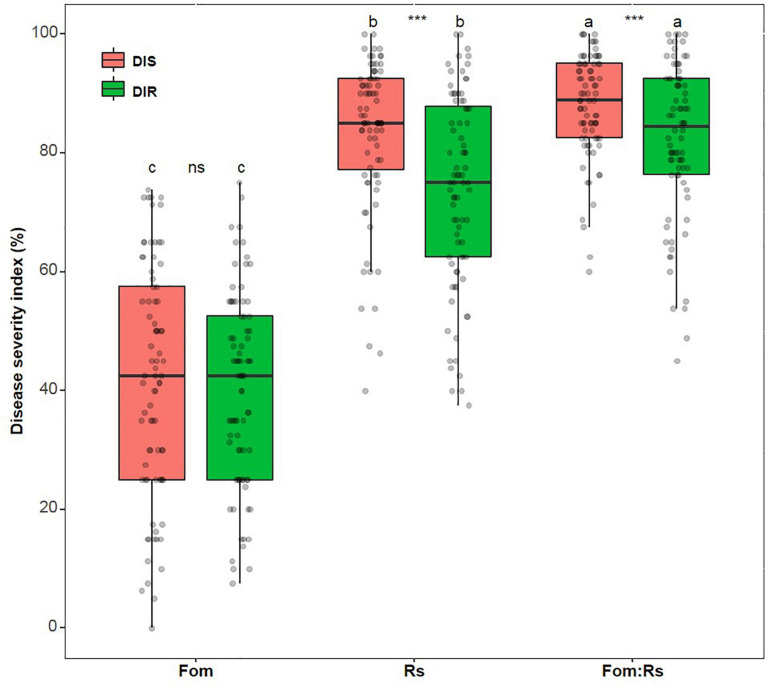
Variations in disease severity among 80 alfalfa varieties single or co-inoculated with Fom and Rs. DIS and DIR, disease index of plant shoot and root, respectively. Fo, single inoculation with Fom; Rs, single inoculation with Rs; Fom:Rs, co-inoculation with Fom and Rs. Plants of the control treatment for companions showed no diseases and data were not included. Boxplots show the medians and 25th and 75th percentiles, with whiskers extending to 1.5 times of the interquartile range, and data presented beyond whiskers represent outliers. Different letters above the bars in the same column indicate significant differences (*p* < 0.001) among treatments according to Fisher’s protected least significant difference (LSD) test. Asterisks between the bars under the same treatment indicate a significant difference (*p* < 0.001) between traits by Student’s *t*-test. ns, not significant between traits.

Across varieties, there was a significant difference in the disease index (DI) of each disease trait among pathogen treatments (*p* < 0.001), with Fom:Rs having the largest effect on disease severity of shoot and root, followed by Rs and Fom (Figure 1 and Supplementary Figure 1). The mean DI of shoot and root under Fom:Rs was 88 and 82%, respectively, which was 3.0- and 2.1-fold of Fom (41 and 40%, respectively) and 1.1- and 1.2-fold of Rs. In addition, the two disease traits showed a significant difference under both Fom:Rs and Rs (*p* < 0.001), with the disease for shoot being more severe than root, while shoot and root had no significant difference under Fom (*p* = 0.22). Among varieties, a variation in disease severity existed for shoot and root under each pathogen treatment (Figure 1; Supplementary Figure 1; and Supplementary Table 2). The DI of shoot (60–100%) and root (45–100%) under Fom:Rs varied 1.7- and 2.2-fold, respectively. There was a 2.5- and 2.7-fold variation in the DI of shoot (40–100%) and root (38–100%) under Rs, respectively, while the DI of shoot and root ranged from 0 to 74% and 8 to 75% (9.5-fold) under Fom, respectively. In several varieties, Fom:Rs led to less severe disease (14–31%) for root than Rs, especially for six varieties (Msv16, Msv19, Msv23, Msv24, Msv36, and Msv80) (Supplementary Figure 1).

### Plant Growth

**FIGURE 2:**
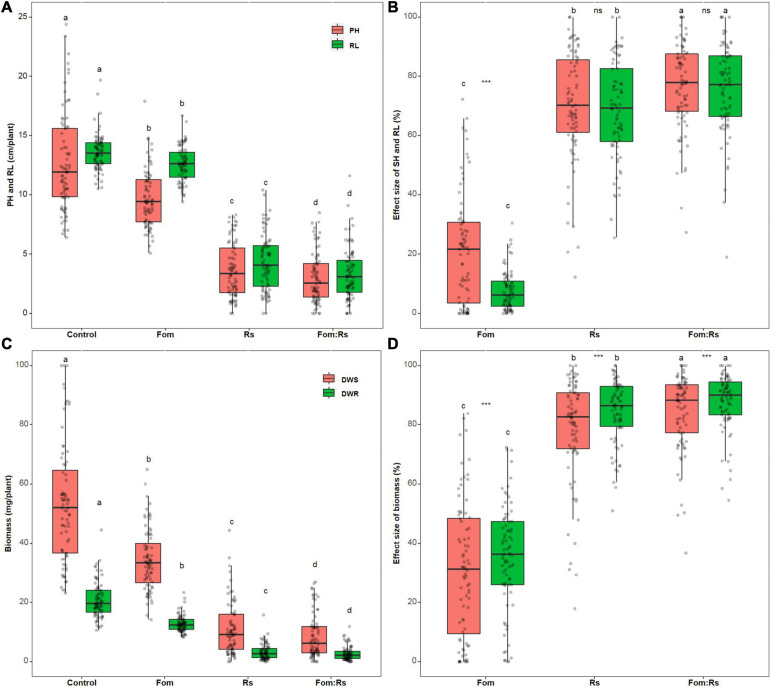
Pathogen effects on plant growth among 80 alfalfa varieties single or co-inoculated with Fom and Rs. **(A)** Variations in plant height (PH) and root length (RL), **(B)** effect sizes of PH and RL, **(C)** variations in dry weight of shoot (DWS) and root (DWR), and **(D)** effect sizes of DWS and DWR. Fom, single inoculation with Fom; Rs, single inoculation with Rs; Fom:Rs, co-inoculation with Fom and Rs. Boxplots show the medians and 25th and 75th percentiles, with whiskers extending to 1.5 times of the interquartile range, and data presented beyond whiskers represent outliers. Different letters above the bars in the same column indicate significant differences (*p* < 0.001) among treatments according to Fisher’s protected LSD test. Asterisks between the bars under the same treatment indicate a significant difference (*p* < 0.001) between traits by Student’s *t*-test. ns, not significant between traits. Three values of DWS larger than 100 (115.1, 145.9, and 119.6 mg/plant for Mvv16, Msv17, and Msv27, respectively) under the control treatment were defined as 100 to maximize resolution. Positive effects (effect size < 0) of pathogen treatment on some traits under Fo (Supplementary Figure 2) were defined as zero to maximize resolution.

There was a significant difference in both plant height and root length across varieties among the pathogen and control treatments (*p* < 0.001) (Figure 2A and Supplementary Table 2). Fom:Rs produced the lowest plant height and root length, followed by Rs and Fom, which was all significantly lower than the control treatment. Across varieties, the effect size of both plant height and root length showed a significant difference among the pathogen treatments (*p* < 0.001) (Figure 2B and Supplementary Table 3). Fom:Rs showed the largest effect on both plant height and root length, followed by Rs and Fom. The mean effect size of plant height and root length under Fom:Rs (76 and 75%, respectively) was 4.2- and 10.0-fold of Fom as well as 1.1- and 1.2-fold of Rs. In addition, under both Fom:Rs and Rs, the effect size of plant height and root length had no significant difference (*p* = 0.31 and 0.36, respectively), but was significantly larger (2.4-fold) than that of root length under Fom. Among varieties, the effect sizes of both plant height and root length showed a variation under each pathogen treatment (Figure 2B and Supplementary Figures 2A,B). Under Fom:Rs, there was a 3.7- and 5.3-fold variation in effect sizes of plant height (27–100%) and root length (19–100%), respectively. The effect sizes of plant height and root length varied 8.1- and 3.9-fold under Rs, respectively, and the effect sizes of plant height and root length under Fom ranged from −46 to 72% and −9 to 30%, respectively.

Biomass traits including shoot and root DW showed a significant difference among the pathogen and control treatments (*p* < 0.001) (Figure 2C and Supplementary Table 2). Fom:Rs produced the lowest shoot DW and root DW, followed by Rs and Fom, which were all significantly lower than the control treatment. Across varieties, the effect size of each biomass trait exhibited a significant difference among the pathogen treatments (*p* < 0.001) (Figure 2D and Supplementary Table 3). Fom:Rs had the largest effect on both biomass traits, followed by Rs and Fom. The mean effect size of shoot DW and root DW under Fom:Rs was 84 and 87%, respectively, which was 2.5- and 2.9-fold of Fom and 1.1- and 1.2-fold of Rs. In addition, the biomass traits showed a significant difference under each pathogen treatment (*p* < 0.001), with root DW always having higher reduction than shoot DW. Among varieties, there was a variation in the effect size of all biomass traits under each pathogen treatment (Figure 2D and Supplementary Figures 2C,D). Fom:Rs caused a 1.8- to 2.7-fold variation in effect sizes of shoot DW (37–100%) and root DW (55–100%), and the effect sizes of shoot DW and root DW under Fom ranged from −46 to 84% and −44 to 72%, respectively.

In addition, Fo showed positive effects on the growth of several varieties (Supplementary Figure 2). Two varieties (Msv60 and Msv68) showed an increase in the four growth traits (5–47%), and six varieties exhibited an increase in both plant height and shoot DW (4–23%), while root length and DW slightly (<14%) reduced in comparison with control treatment. It was also shown that Fom:Rs led to a smaller negative effect on the growth of several varieties than Rs, especially for five varieties (Msv19, Msv23, Msv24, Msv36, and Msv80) that had 12% to 53% less reductions in all the five growth traits (Supplementary Figure 2).

### Biomass Allocation

**FIGURE 3:**
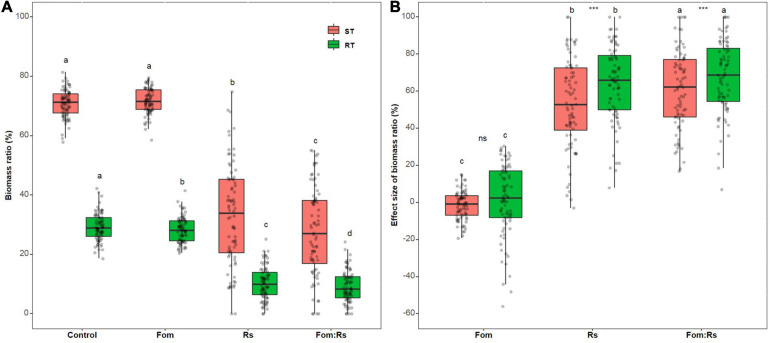
Pathogen effects on biomass allocations among 80 alfalfa varieties single or co-inoculated with Fom and Rs. **(A)** Variations in shoot biomass ratio (ST) and root biomass ratio (RT) and **(B)** effect sizes of ST and RT. Fom, single inoculation with Fom; Rs, single inoculation with Rs; Fom:Rs, co-inoculation with Fom and Rs. Boxplots show the medians and 25th and 75th percentiles, with whiskers extending to 1.5 times of the interquartile range, and data presented beyond whiskers represent outliers. Different letters above the bars in the same column indicate significant differences (*p* < 0.001) among treatments according to Fisher’s protected LSD test. Asterisks between the bars under the same treatment indicate a significant difference (*p* < 0.001) between traits by Student’s *t*-test. ns, not significant between traits.

There was a significant difference in the biomass ratio of ST and RT across varieties among the pathogen and control treatments (*p* < 0.001) (Figure 3A and Supplementary Table 2). Fom:Rs produced the lowest biomass ratio of ST and RT, followed by Rs and Fom, which were all significantly lower than the control treatment (except ST between Fo and control). Across varieties, the effect size of each biomass ratio showed a significant difference among the pathogen treatments (*p* < 0.001) (Figure 3B and Supplementary Table 3). Fom:Rs had the largest effect on ST and RT, followed by Rs and Fom. The mean effect size of ST and RT under Fom:Rs was 61 and 68%, respectively, which was 13.1- and 51.2-fold of Fom, and 1.1- and 1.2-fold of Rs. The biomass ratios showed a significant difference under both Fom:Rs and Rs (*p* < 0.001), with RT always having larger reduction than ST while there was no significant difference under Fom (*p* = 0.34). Among varieties, the effect sizes of each biomass ratio showed a variation under each pathogen treatment (Figure 3B and Supplementary Figure 3). Fom:Rs had a 5.9- to 12.7-fold variation in effect size of ST (17–100%) and RT (8–100%), and the effect size of ST and RT under Fo ranged from −19 to 15% and −56 to 30%, respectively.

Interestingly, Fo even showed positive effects on biomass ratios of several varieties, especially RT (Supplementary Figure 3). Mvv27 exhibited a 56% increase in RT while the others had an increase of lower than 50%. In addition, Fom:Rs led to a smaller negative effect on biomass ratios of several varieties than Rs, especially for five varieties (Msv19, Msv23, Msv24, Msv36, and Msv80) that had 11–69% less reductions in biomass allocation traits (Supplementary Figure 3).

### Root Morphology

**FIGURE 4:**
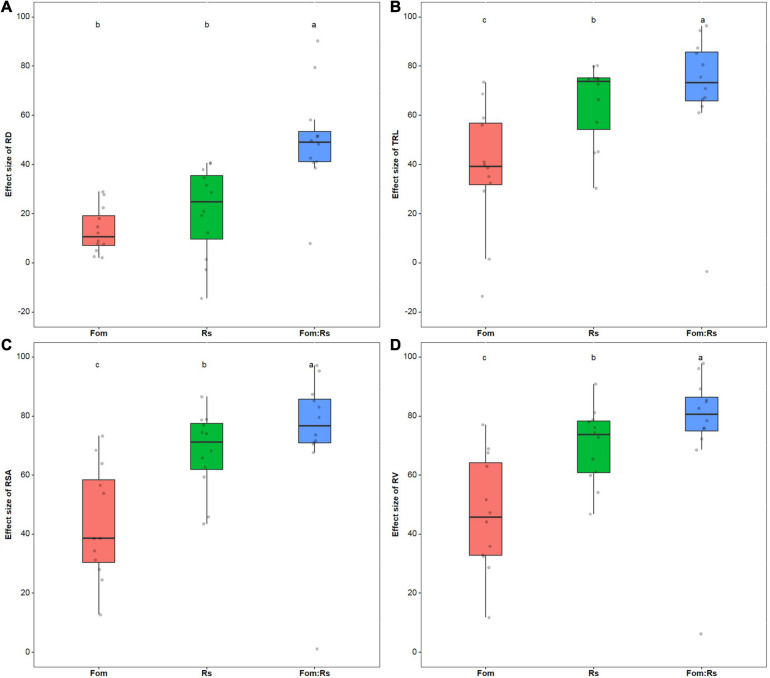
Pathogen effects on root morphology among 12 alfalfa varieties single or co-inoculated with Fom and Rs. Effect sizes of **(A)** root diameter (RD), **(B)** total root length (TRL), **(C)** root surface area (RSA), and **(D)** root volume (RV). Fom, single inoculation with Fom; Rs, single inoculation with Rs; Fom:Rs, co-inoculation with Fom and Rs. Boxplots show the medians and 25th and 75th percentiles, with whiskers extending to 1.5 times of the interquartile range, and data presented beyond whiskers represent outliers. Different letters above the bars indicate significant differences (*p* < 0.001) among treatments according to Fisher’s protected LSD test at *p* = 0.05.

Across varieties, root morphology traits including RD, TRL, RSA, and RV were all significantly reduced under each pathogen treatment compared with the control treatment (*p* < 0.001) (Supplementary Figure 4). The effect size of RD had no significant difference (*p* = 0.29) between the single treatments of Fom and Rs (a mean of 13 and 21%, respectively), but significantly lower (*p* < 0.001) than that of Fom:Rs (a mean of 50%) (Figure 4A). Moreover, there was a significant difference (*p* < 0.001) in the effect size of TRL, RSA, and RV among the pathogen treatments, and Fom:Rs showed the largest effect on all traits (a mean of 71, 74, and 76% for TRL, RSA, and RV, respectively), followed by Rs and Fom (Figures 4B–D). In addition, Rs showed slightly positive effects on RD of two varieties (Msv62 and Msv26) with an increase of 3 and 14%, respectively, and Fom and Fom:Rs resulted in a slight increase in TRL for one variety Msv62 (13 and 3%, respectively).

### Correlations Among Pathogen Effect Size of Traits

Both similar and varied relationships showed among the effect sizes of growth and biomass allocation traits with disease traits upon varying pathogen infection (Supplementary Table 4). DIS and DIR were positively correlated with each other under different pathogen treatments (*p* < 0.001, *r* = 0.77–0.89). The effect size of three growth traits including PH, DWS, and DWR and the biomass allocation trait ST were all positively correlated with DIS and DIR under different pathogen treatments (*p* < 0.001, *r* = 0.45–0.74, 0.66–0.90, and 0.67–0.84 for Fom, Rs, and Fom:Rs, respectively). In addition, the effect size of RL had no significant correlation with DIS and DIR under Fom (*p* = 0.76 and 0.85, respectively) but was positively correlated with DIS and DIR under both Rs and Fom:Rs (*p* < 0.001, *r* = 0.77–0.87). The effect size of biomass allocation trait RT was negatively correlated with DIS and DIR under Fom (*p* < 0.001, *r* = −0.49 and −0.47, respectively), but positively correlated with DIS and DIR under both Rs and Fom:Rs (*p* < 0.001, *r* = 0.60–0.79).

### Varietal Response Patterns

**FIGURE 5:**
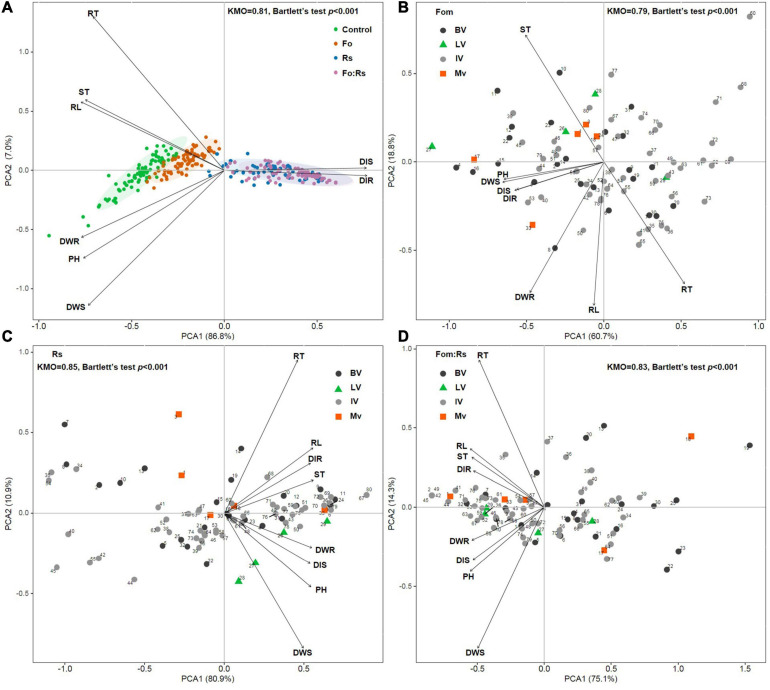
Principal component analysis (PCA) on all plant traits and pathogen effect sizes for 80 alfalfa varieties single or co-inoculated with Fom and Rs. **(A)** PCA of all plant traits under the control and three pathogen treatments. PCA on pathogen effect sizes of plant traits under **(B)** single inoculation with Fom (Fom), **(C)** single inoculation with Rs (Rs), and **(D)** co-inoculation with Fom and Rs (Fom:Rs). Biplot vectors are trait factor loadings, whereas the position of each variety is shown. Kaiser–Meyer–Olkin (KMO) measure of sampling adequacy and Bartlett’s test of sphericity are shown. The study includes 75 *M. sativa* subsp. *sativa* (Ms) varieties consisting of 24 domestic bred (BV, circles in black), 4 local varieties (LV, triangles) in China, and 47 introduced varieties overseas (IV, circles in gray), and 5 *M. sativa* subsp. *varia* domestic bred varieties (Mv, squares). Numbers of varieties shown in panels **(B–D)** corresponds to those shown in Supplementary Table 1.

Principal component analysis based on disease traits as well as growth and biomass allocation traits among 80 alfalfa varieties showed that varieties under each pathogen treatment were clustered and separated from the control treatment (Figure 5A). Varieties under Fom:Rs and Rs were closely related, being distinctly separated from varieties under Fom. PCA based on disease traits and pathogen effect sizes of growth and biomass allocation among varieties showed no clear patterns according to the varietal type (domestic, local, or introduced variety) upon varying pathogen infection (Figures 5B–D). Varieties of *M. sativa* subsp. *sativa* that belonged to the domestic, local, or introduced type were not closely related, and the five domestic bred varieties of *M. sativa* subsp. *varia* were also not closely related.

**FIGURE 6:**
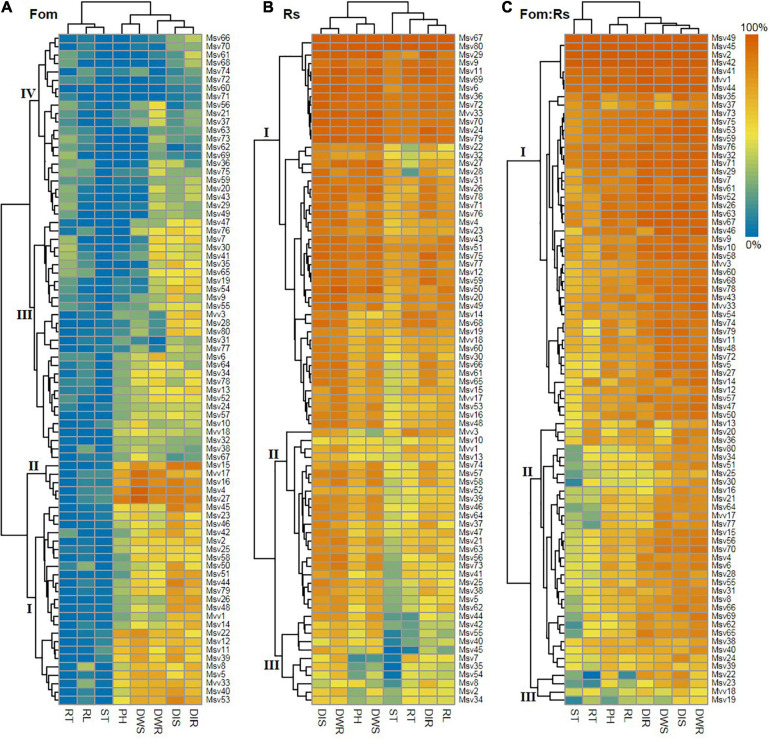
Hierarchical clustering of 80 alfalfa varieties based on variations in pathogen effect sizes of all plant traits single and co-inoculated with Fom and Rs. **(A)** Single inoculation with Fom (Fom), **(B)** single inoculation with Rs (Rs), and **(C)** co-inoculation with Fom and Rs (Fom:Rs). Labels on the dendrogram (I, II, III, and IV) represent the main groups. The heat map shows variations in pathogen effect sizes of different traits. The color gradient range (0–100%) is shown on the top right corner. Positive effects (effect size < 0) of pathogen treatment on some traits (Supplementary Figures 1–4) were defined as zero to maximize resolution. DIS and DIR, disease index of plant shoot and root, respectively; PH, plant height; RL, root length; DWS and DWR, dry weight of shoot and root, respectively; ST, shoot biomass ratio; RT, root biomass ratio.

Hierarchical clustering based on variations in disease severity as well as pathogen effects on growth and biomass allocation among 80 alfalfa varieties revealed differential host response patterns upon varying pathogen infections (Figure 6). Varieties were also clustered into four main groups under Fo that involved 29 varieties (36%) in groups I and II most with relatively high effect sizes, 22 varieties (28%) in group IV having relatively low effect sizes, and the remaining ones in group III with moderate effect sizes. Under Rs, varieties were separated into three main groups with 47 varieties (59%) in group I that had relatively high effect sizes, 11 varieties (14%) in group III having relatively low effect sizes, and the remaining ones in group II having moderate effect sizes. Under Fom:Rs, there were three main groups with 46 varieties (58%) in group I that had relatively high effect sizes, only 4 varieties (5%) in group III having relatively low effect sizes, and the remaining ones in group II having moderate effect sizes.

### Comprehensive Resistance Evaluation

The comprehensive resistances of the 80 varieties were ranked based on the membership function value that defined the weight of different plant traits including disease, growth, and biomass allocation traits (Table 1 and Supplementary Figure 5). Varieties that were most resistant (ranked in top 10) to either single infection by Fom or Rs were not ranked in the top 10 under the co-infection. Under Fom, the top 10 varieties were the introduced varieties Msv60, Msv71, Msv72, Msv70, Msv62, Msv69, Msv66, Msv74, Msv63, and Msv68, with the DI of disease traits ranging from 0 to 33% and effect sizes of biomass traits ranging from −46 to 23%. Among these, two varieties (Msv70 and Msv69) and another two varieties (Msv71 and Msv63) were ranked within the bottom 10 under Rs and Fom:Rs, respectively, with DI >85% and effect sizes of biomass traits >78%. The top 10 varieties to Rs involved seven introduced varieties (Msv35, Msv40, Msv54, Msv45, Msv34, Msv55, and Msv42) and three domestic varieties (Msv8, Msv7, and Msv2), with the DI of disease traits ranging from 38 to 75% and effect sizes of biomass traits ranging from 12 to 73%. In addition, the three varieties (Msv45, Msv2, and Msv42) ranked within the bottom 10 showed no resistance to Fom:Rs, with plants dead. The top 10 ranked varieties under Fom:Rs included eight domestic varieties (Msv19, Msv23, Msv25, Mvv18, Msv22, Msv30, Msv16, and Msv24) and two introduced varieties (Msv39 and Msv64), with the DI of disease traits ranging from 45 to 85% and effect sizes of biomass traits ranging from 19 to 82%. Among these, three varieties (Msv22, Msv39, and Msv16) under Fom and one variety (Msv24) under Rs ranked within the bottom 10, with the DI ranging from 31 to 77% and effect sizes of biomass traits all >95%.

**TABLE 1:** Comprehensive resistance ranking of the 80 alfalfa varieties to Fom and Rs under either single or co-infection based on the membership function value (*D*_*v*_).

**Variety no.**	**Fom*^*a*^***		**Rs*^*b*^***		**Fom:Rs*^*c*^***		**Variety no.**	**Fom*^*a*^***		**Rs*^*b*^***		**Fom:Rs*^*c*^***	
	***D*_*v*_**	**Rank**	***D*_*v*_**	**Rank**	***D*_*v*_**	**Rank**		***D*_*v*_**	**Rank**	***D*_*v*_**	**Rank**	***D*_*v*_**	**Rank**
Mvv1	0.602	44	0.460	21	0.051	75	Msv41	0.582	48	0.493	18	0.067	74
Msv2	0.500	61	**0.660**	**9**	0.000	77	Msv42	0.479	63	**0.646**	**10**	0.000	78
Mvv3	0.623	38	0.478	20	0.244	48	Msv43	0.742	17	0.174	62	0.198	55
Msv4	0.158	79	0.274	52	0.399	27	Msv44	0.445	65	0.558	13	0.045	76
Msv5	0.390	72	0.525	14	0.299	37	Msv45	0.412	70	**0.750**	**5**	0.000	79
Msv6	0.601	45	0.103	71	0.364	32	Msv46	0.508	60	0.404	29	0.170	60
Msv7	0.563	53	**0.747**	**6**	0.141	63	Msv47	0.692	27	0.415	26	0.279	42
Msv8	0.436	66	**0.761**	**4**	0.393	29	Msv48	0.519	58	0.310	47	0.252	47
Msv9	0.693	26	0.113	68	0.211	53	Msv49	0.774	14	0.212	57	0.000	80
Msv10	0.579	49	0.631	11	0.187	57	Msv50	0.546	57	0.161	65	0.290	40
Msv11	0.389	73	0.075	78	0.275	43	Msv51	0.485	62	0.150	67	0.510	12
Msv12	0.432	67	0.168	64	0.324	35	Msv52	0.559	54	0.499	16	0.138	66
Msv13	0.594	46	0.565	12	0.494	15	Msv53	0.381	74	0.371	37	0.113	69
Msv14	0.574	51	0.315	46	0.291	39	Msv54	0.621	39	**0.766**	**3**	0.237	50
Msv15	0.317	76	0.380	35	0.392	30	Msv55	0.638	33	**0.686**	**8**	0.427	23
Msv16	0.214	78	0.359	38	**0.551**	**8**	Msv56	0.719	21	0.402	30	0.342	33
Mvv17	0.252	77	0.401	31	0.497	14	Msv57	0.636	34	0.352	40	0.258	46
Mvv18	0.621	40	0.324	44	**0.692**	**4**	Msv58	0.564	52	0.375	36	0.172	58
Msv19	0.629	37	0.339	42	**0.897**	**1**	Msv59	0.736	19	0.169	63	0.136	67
Msv20	0.688	28	0.197	59	0.426	24	Msv60	**0.968**	**1**	0.325	43	0.241	49
Msv21	0.721	20	0.416	25	0.481	17	Msv61	0.799	12	0.310	49	0.170	59
Msv22	0.365	75	0.396	32	**0.643**	**5**	Msv62	**0.871**	**5**	0.515	15	0.473	18
Msv23	0.516	59	0.310	48	**0.728**	**2**	Msv63	**0.831**	**9**	0.409	28	0.109	71
Msv24	0.632	36	0.089	75	**0.550**	**9**	Msv64	0.647	32	0.410	27	**0.521**	**10**
Msv25	0.553	56	0.491	19	**0.714**	**3**	Msv65	0.609	43	0.322	45	0.403	25
Msv26	0.559	55	0.211	58	0.159	61	Msv66	**0.842**	**7**	0.288	51	0.399	26
Msv27	0.125	80	0.294	50	0.299	38	Msv67	0.701	24	0.000	79	0.129	68
Msv28	0.683	29	0.359	39	0.451	20	Msv68	**0.822**	**10**	0.236	53	0.217	52
Msv29	0.740	18	0.103	72	0.140	64	Msv69	**0.855**	**6**	0.083	77	0.396	28
Msv30	0.633	35	0.344	41	**0.626**	**6**	Msv70	**0.874**	**4**	0.102	73	0.334	34
Msv31	0.818	11	0.221	55	0.431	22	Msv71	**0.951**	**2**	0.224	54	0.091	73
Msv32	0.698	25	0.437	22	0.092	72	Msv72	**0.902**	**3**	0.112	69	0.285	41
Mvv33	0.429	68	0.103	70	0.195	56	Msv73	0.784	13	0.394	34	0.144	62
Msv34	0.576	50	**0.721**	**7**	0.518	11	Msv74	**0.840**	**8**	0.396	33	0.259	45
Msv35	0.611	41	**0.773**	**1**	0.211	54	Msv75	0.680	30	0.157	66	0.139	65
Msv36	0.751	15	0.090	74	0.382	31	Msv76	0.610	42	0.215	56	0.112	70
Msv37	0.715	22	0.425	24	0.319	36	Msv77	0.748	16	0.191	61	0.507	13
Msv38	0.714	23	0.497	17	0.458	19	Msv78	0.587	47	0.193	60	0.222	51
Msv39	0.410	71	0.431	23	**0.591**	**7**	Msv79	0.453	64	0.088	76	0.272	44
Msv40	0.419	69	**0.770**	**2**	0.448	21	Msv80	0.659	31	0.000	80	0.489	16

*A larger *D*_*v*_ value indicates that the resistance of the variety is higher while a smaller value indicates that the resistance is lower. The varieties ranked in the top 10 (in bold) were considered as the most resistant varieties under each pathogen treatment. *^*a*^*Fom, single infection by Fom. *^*b*^*Rs, single infection by Rs. *^*c*^*Fom:Rs, co-infection by Fom and Rs.*

## Discussion

This study provides the first information on the effects of co-infection by Fom and Rs on disease severity and growth performance of diverse alfalfa varieties as well as the host resistance responses under co-infection. Across varieties, the co-infection led to increased disease severity as well as reduced plant growth and biomass allocation compared with either single infection. In addition, root morphology was more strongly altered by the co-infection. The reductions in different growth traits (plant height, shoot biomass, and root biomass) and shoot biomass allocation was all positively correlated with disease severity under both single and co-infection. Furthermore, varieties that were most resistant to either single infection were not effective to the co-infection.

Across varieties, the co-infection by Fom and Rs resulted in significantly increased disease severity along with reduced growth and biomass allocation compared with either single infection. This is consistent with previous findings on other legume crops that the co-infection of plants with soil-borne pathogens had a significant increase in disease epidemic compared with single infection. For example, the co-infection by Fo and *Pythium ultimum* led to a significant increase in disease development of root rot in pea compared with single infection (Kerr, 1963), and root disease was more severe when snap bean was co-infected by *P. ultimum* and *Aphanomyces euteiches* (Pfender and Hagedorn, 1982). Likewise, the co-inoculation of *A. euteiches* and *Fusarium solani* resulted in an increase of root rot in pea (Peters and Grau, 2002). In addition to increased disease severity, the reduction in growth traits under co-infection was significantly higher than that of Fom or Rs. Previous studies have shown reductions in plant growth such as plant height, root length, and root or shoot biomass of alfalfa against single infection by Fom and Rs (Guo et al., 2015; Xin et al., 2016). Reduced plant growth has also been reported on alfalfa to the single infection by *Pythium* spp., tomato against Fo, and soybean to other *Fusarium* species (Larkin et al., 1995, 1996; Arias et al., 2013; Morauf and Steinkellner, 2015). This study also found that varieties infected by Rs were closely related to the co-infection and were distinctly separated from those infected by Fom (Figure 6A). These findings indicate that Fom and Rs can infect roots simultaneously that produce more devastating disease epidemics in alfalfa.

The enhanced effects of Fom and Rs co-infection on disease severity and plant growth may be due to their different action locations within root tissues after penetrating through the root epidermis. Fo enters through the root cortex to xylem vessels where proliferation, growth, and spread occur without damaging cortex tissues (Fang et al., 2012; Fang and Barbetti, 2014; Gordon, 2017) while Rs damages the intracellular cement of root cortex tissues where cells dissolve without affecting xylem vessels (Ajayi-Oyetunde and Bradley, 2018; Batnini et al., 2020). It is likely that the two root pathogens work cooperatively with Fo acting in xylem vessels and Rs acting in cortex tissues, which result in enhanced negative effects on plant growth when plants were co-infected. We also found that the co-infection resulted in reduced disease severity and growth performance compared with one single infection by Rs in several varieties, especially for varieties Msv19, Msv23, Msv24, Msv36, and Msv80 that had 11–69% less severe disease as well as less reductions in growth traits and biomass ratios. It has been reported that the co-infection by soil-borne pathogens *Pythium* and *Fusarium* species did not result in more severe root disease in soybean (Lerch-Olson and Robertson, 2020). It is highlighted that different modes of pathogen interactions may occur when plants are co-infected, where different pathogens can act as cooperative interactions or have competitive interactions (Abdullah et al., 2017). Therefore, in addition to cooperative interaction, these findings suggest that Fo and Rs may also have competitive interaction when plants are co-infected, with the modes of pathogen interactions showing varietal difference.

Disease severity for shoot and root was found positively correlated with each other against either single or co-infection by Fom and Rs; besides, the reductions in plant height, shoot biomass, root biomass, and shoot biomass allocation were all positively correlated with disease severity. The positive correlations between growth reduction with disease severity have been previously demonstrated in other pasture crops against soil-borne pathogens, as shown in subterranean clover infected by *Phytophthora clandestina* (You and Barbetti, 2017). Our study suggests that shoot symptoms can be a consistent indicator of root symptoms and the coordination of different growth traits and shoot biomass allocation in response to Fom and Rs under either single or co-infection.

Root morphological changes represent an adaptive response of plants that evolve to maintain water and nutrient supply under the challenge of soil-borne pathogens (Arias et al., 2013; Buhtz et al., 2017). Root morphological traits, including RD, total length, surface area, and volume across varieties, were all significantly reduced against either single or co-infection by Fom and Rs. Reductions in such root morphology traits have been revealed in alfalfa and other crops to single infection by soil-borne pathogens. For example, alfalfa seedlings infected by *Pythium* spp., particularly *P. ultimum* and *Pythium irregulare*, showed reductions in root growth and changes in root system architecture (Larkin et al., 1995, 1996). In tomato, Fo caused decreased root length, root weight, RSA, and RV (Morauf and Steinkellner, 2015). The root length and surface area of tomato were reduced in response to the infection by *Verticillium dahliae* (Buhtz et al., 2017) or *Pythium aphanidermatum* (Schwarz and Grosch, 2003). In addition, the single infection by *Fusarium* spp. such as *F. graminearum*, *F. proliferatum*, and *F. virguliform* led to reductions in RD, TRL, surface area, and RV in soybean (Arias et al., 2013). We also found that the co-infection by Fom and Rs led to higher reductions in root morphology traits than either single infection, which further indicated the roles of root morphological traits in co-infection by soil-borne pathogens in addition to a single pathogen infection. In addition, we found that root biomass was more reduced than shoot biomass under either single or co-infection by Fom and Rs, highlighting the roles of the two pathogens in root disease complex of alfalfa.

Comprehensive resistance evaluations on different varieties using the membership function method that considered the weight of all disease, growth, and biomass allocation traits enabled the identification of most resistant varieties to each pathogen treatment. We found that the varieties most resistant to either single infection were not the most resistant varieties to the co-infection, with some being the most susceptible to the co-infection. For varieties that were most resistant to Fom, varieties Msv71 and Msv63 were most susceptible to the co-infection (ranked in the bottom 10 based on membership function value). Among the top 10 varieties most resistant to Rs, varieties Msv45, Msv2, and Msv42 were the most susceptible to the co-infection with all plants dead. This confirmed the opinion that plants with resistance to a single pathogen were not effective against the co-occurrence of different pathogens (Tollenaere et al., 2016; Wille et al., 2019). In addition, varieties most resistant to the single infection by either Fom or Rs were different. Such genotypic differences in disease resistance to single infection by different soil-borne pathogens have been shown in other legume crops such as subterranean clover (You et al., 2020). Furthermore, hierarchical clustering found differential response patterns among varieties upon co-infection compared with either single infection, and most varieties were highly susceptible to the co-infection. Taken together, our findings suggest that the co-infection by Fom and Rs altered disease resistance response to either single infection and implies that no any individual variety was resistant to both pathogens singly and co-infected, which further highlights the difficulties in effective control of the soil-borne pathogens in alfalfa.

This study also highlights the potential to develop alfalfa varieties that are both high in disease resistance and growth performance under the challenge of soil-borne pathogens. We identified several varieties with increased growth under the single infection by Fom. It is noteworthy that varieties Msv60 and Msv68 showed an increase in all growth traits (5–47%) and varieties Msv71, Msv72, Msv62, and Msv69 had an increase in both plant height and shoot DW (4–23%), being slightly reduced in root length and DW (<14%). High forage yield is the major focus in alfalfa breeding, and yield increases have been attributed in part to improved disease resistance (Lamb et al., 2000; Hakl et al., 2017; Shi et al., 2017). The varieties with resistance to the single infection of Fom or Rs indicate their potential as resistance donors for alfalfa breeding. Further studies to explore genes and mechanisms underlying the resistance of varieties to the single infection by Fom or Rs will enable pyramiding different resistance genes into alfalfa varieties for conferring effective resistance to co-occurring pathogens.

In conclusion, for the first time, this study demonstrates that the co-infection by Fom and Rs alters disease resistance responses among diverse alfalfa varieties. This study also reveals the coordination of different growth traits and shoot biomass allocation when plants are co-infected by Fom and Rs, and altered root morphology is employed by plants in response to the co-infection. This study provides valuable information for developing alfalfa varieties with resistance against different soil-borne pathogens that occur as pathogen complexes in the field, offering future opportunities to manage in an effective and sustainable way. Varieties with resistance to the single infection of Fom or Rs are of great value for further studies to explore genes and mechanisms conferring disease resistance in alfalfa. This study also highlights the importance to study mechanisms underlying the co-infection and interactions of different soil-borne pathogens with plant hosts, rather than always focusing on a single pathogen, if we are to make significant progress in the management of soil-borne pathogens.

## Data Availability Statement

The datasets presented in this study can be found in online repositories. The names of the repository/repositories and accession number(s) can be found below: https://www.ncbi.nlm.nih.gov/genbank/, MW036290; https://www.ncbi.nlm.nih.gov/genbank/, MW560899; and https://www.ncbi.nlm.nih.gov/genbank/, MW036291.

## Author Contributions

XF designed the study. CZ, TD, BY, XJ, and YW conducted the experiments. XF and ZW analyzed the data. XF, JP, and LM wrote the manuscript. ZN supervised the project. All authors contributed to the article and approved the submitted version.

## Conflict of Interest

The authors declare that the research was conducted in the absence of any commercial or financial relationships that could be construed as a potential conflict of interest.

## Supplementary Material

Supplementary Figure 1 Disease severity of 80 alfalfa varieties that were single or co-inoculated with Fom and Rs.

Supplementary Figure 2 Pathogen effects on plant growth of 80 alfalfa varieties that were single or co-inoculated with Fom and Rs.

Supplementary Figure 3 Pathogen effects on biomass allocation of 80 alfalfa varieties that were single or co-inoculated with Fom and Rs.

Supplementary Figure 4 Supplementary FiVariations in root morphology among 12 alfalfa varieties that were single or co-inoculated with Fom and Rs.

Supplementary Figure 5 Supplementary FiComprehensive resistance ranking of the 80 alfalfa varieties to Fo and Rs under either single or co-infection based on the membership function value (*D*_*v*_).

Supplementary Table 1 Details of alfalfa (*Medicago sativa* subsp. *sativa* and *M. sativa* subsp. *varia*) varieties used in this study.

Supplementary Table 2 Statistical single effect and interaction for the two-way analysis of variance of variety and pathogen treatment on the disease severity, growth and biomass allocation traits.

Supplementary Table 3 Statistical single effect and interaction for the two-way analysis of variance of variety and pathogen treatment on the pathogen effect sizes of growth and biomass allocation traits.

Supplementary Table 4 Pearson’s correlation matrix for the disease severity index and pathogen effect sizes of growth and biomass allocation traits across 80 alfalfa varieties that were single or co-inoculated with Fom and Rs.

## References

[B1] Abdullah A. S. Moffat C. S. Lopez-Ruiz F. J. Gibberd M. R. Hamblin J. Zerihun A. (2017). Host-multi-pathogen warfare: pathogen interactions in co-infected plants. *Front. Plant Sci.* 8:1806. 10.3389/fpls.2017.01806 29118773 PMC5660990

[B2] Ajayi-Oyetunde O. O. Bradley C. A. (2018). *Rhizoctonia solani*: taxonomy, population biology and management of *Rhizoctonia* seedling disease of soybean. *Plant Pathol.* 67 3–17. 10.1111/ppa.12733

[B3] Anderson J. P. Lichtenzveig J. Oliver R. P. Singh K. B. (2013). *Medicago truncatula* as a model host for studying legume infecting *Rhizoctonia solani* and identification of a locus affecting resistance to root canker. *Plant Pathol.* 62 908–921. 10.1111/j.1365-3059.2012.02694.x

[B4] Arias M. M. D. Leandro L. F. Munkvold G. P. (2013). Aggressiveness of *Fusarium* species and impact of root infection on growth and yield of soybeans. *Phytopathology* 103 822–832. 10.1094/phyto-08-12-0207-r 23514263

[B5] Bai Z. Ma W. Ma L. Velthof G. L. Wei Z. Havlík P. (2018). China’s livestock transition: driving forces, impacts, and consequences. *Sci. Adv.* 4:eaar8534. 10.1126/sciadv.aar8534 30035221 PMC6051741

[B6] Batnini M. Fernández Del-Saz N. Fullana-Pericàs M. Palma F. Haddoudi I. Mrabet M. (2020). The alternative oxidase pathway is involved in optimizing photosynthesis in *Medicago truncatula* infected by *Fusarium oxysporum* and *Rhizoctonia solani*. *Physiol. Plant.* 169 600–611. 10.1111/ppl.13080 32108952

[B7] Berrocal-Lobo M. Molina A. (2008). Arabidopsis defense response against *Fusarium oxysporum*. *Trends Plant Sci.* 13 145–150. 10.1016/j.tplants.2007.12.004 18289920

[B8] Buhtz A. Hohe A. Schwarz D. Grosch R. (2017). Effects of *Verticillium dahliae* on tomato root morphology considering plant growth response and defence. *Plant Pathol.* 66 667–676. 10.1111/ppa.12595

[B9] Cao L. Liu H. Zhao S. (2011). Effect of soil conditioners on water stability of soil aggregates and its mechanisms in loessal soil. *Sci. Soil Water Conserv.* 9 37–41.

[B10] Chen H. Zeng Y. Yang Y. Huang L. Tang B. Zhang H. (2020). Allele-aware chromosome-level genome assembly and efficient transgene-free genome editing for the autotetraploid cultivated alfalfa. *Nat. Commun.* 11 1–11. 32427850 10.1038/s41467-020-16338-x PMC7237683

[B11] de Mendiburu F. (2020). *Agricolae: Statistical Procedures for Agricultural Research. R Package Version 1.3-3.*

[B12] Dean R. Van Kan J. A. L. Pretorius Z. A. Hammond-Kosack K. E. Di Pietro A. Spanu P. D. (2012). The top 10 fungal pathogens in molecular plant pathology. *Mol. Plant Pathol.* 13 414–430. 10.1111/j.1364-3703.2011.00783.x 22471698 PMC6638784

[B13] Drizou F. Graham N. S. Bruce T. J. A. Ray R. V. (2017). Development of high-throughput methods to screen disease caused by *Rhizoctonia solani* AG 2-1 in oilseed rape. *Plant Methods* 13:45. 28572833 10.1186/s13007-017-0195-1 PMC5450255

[B14] Edel-Hermann V. Lecomte C. (2019). Current status of *Fusarium oxysporum* formae speciales and races. *Phytopathology* 109 512–530. 10.1094/phyto-08-18-0320-rvw 30461350

[B15] Fang X. Barbetti M. J. (2012). Reduced severity and impact of *Fusarium* wilt on strawberry by manipulation of soil pH, soil organic amendments and crop rotation. *Eur. J. Plant Pathol.* 134 619–629. 10.1007/s10658-012-0042-1

[B16] Fang X. Finnegan P. M. Barbetti M. J. (2013). Wide variation in virulence and genetic diversity of binucleate *Rhizoctonia* isolates associated with root rot of strawberry in Western Australia. *PLoS One* 8:e55877. 10.1371/journal.pone.0055877 23405226 PMC3566113

[B17] Fang X. Phillips D. Li H. Sivasithamparama K. Barbetti M. J. (2011). Comparisons of virulence of pathogens associated with crown and root diseases of strawberry in Western Australia with special reference to the effect of temperature. *Sci. Hortic.* 131 39–48. 10.1016/j.scienta.2011.09.025

[B18] Fang X. Zhang C. X. Nan Z. B. (2019). Research advances in *Fusarium* root rot of alfalfa (*Medicago sativa*). *Acta Pratacult. Sin.* 28 169–183.

[B19] Fang X. Barbetti M. J. (2014). Differential protein accumulations in isolates of the strawberry wilt pathogen *Fusarium oxysporum* f. sp. *fragariae* differing in virulence. *J. Proteomics* 108 223–237. 10.1016/j.jprot.2014.05.023 24907490

[B20] Fang X. Kuo J. Finnegan P. M. Barbetti M. J. (2012). Comparative root colonisation of strawberry cultivars Camarosa and Festival by *Fusarium oxysporum* f. sp. *fragariae*. *Plant Soil* 358 75–89. 10.1007/s11104-012-1205-8

[B21] Fravel D. Olivain C. Alabouvette C. (2003). *Fusarium oxysporum* and its biocontrol. *New Phytol.* 157 493–502. 33873407 10.1046/j.1469-8137.2003.00700.x

[B22] Gill U. S. Uppalapati S. R. Gallego-Giraldo L. Ishiga Y. Dixon R. A. Mysore K. S. (2018). Metabolic flux towards the (iso) flavonoid pathway in lignin modified alfalfa lines induces resistance against *Fusarium oxysporum* f. sp. *medicaginis*. *Plant Cell Environ.* 41 1997–2007. 29047109 10.1111/pce.13093

[B23] Gordon T. R. (2017). *Fusarium oxysporum* and the *Fusarium* wilt syndrome. *Annu. Rev. Phytopathol.* 55 23–39. 10.1146/annurev-phyto-080615-095919 28489498

[B24] Guo Y. X. Zhang M. Guan Y. Z. Liu F. Guo Z. P. Zhang H. R. (2015). Pathogenicity of *Rhizoctonia solani* to alfalfa seedlings and disease resistance of alfalfa varieties. *Acta Pratacult. Sin.* 24 72–81.

[B25] Haas D. Défago G. (2005). Biological control of soil-borne pathogens by fluorescent pseudomonads. *Nat. Rev. Microbiol.* 3 307–319. 10.1038/nrmicro1129 15759041

[B26] Hakl J. Pisarčik M. Hrevušová Z. Šantrůček J. (2017). In-field lucerne root morphology traits over time in relation to forage yield, plant density, and root disease under two cutting managements. *Field Crops Res.* 213 109–117. 10.1016/j.fcr.2017.07.017

[B27] Hane J. K. Anderson J. P. Williams A. H. Sperschneider J. Singh K. B. (2014). Genome sequencing and comparative genomics of the broad host-range pathogen *Rhizoctonia solani* AG8. *PLoS Genet.* 10:e1004281. 10.1371/journal.pgen.1004281 24810276 PMC4014442

[B28] Huang N. Sun X. B. Wang T. M. Lu X. S. (2013). Evaluation of resistance to *Fusarium oxysporum* and preliminary screening of check varieties of resistant evaluation from 62 alfalfa cultivars. *Chin. J. Grassland* 35 12–17.

[B29] Kassambara A. Mundt F. (2020). *Factoextra: Extract and Visualize the Results Of Multivariate Data Analyses. R Package Version 1.0.7.*

[B30] Kerr A. (1963). The root rot-*Fusarium* wilt complex of peas. *Aust. J. Biol. Sci.* 16 55–69. 10.1071/bi9630055

[B31] Khoury C. K. Bjorkman A. D. Dempewolf H. Ramirez-Villegas J. Guarino L. Jarvis A. (2014). Increasing homogeneity in global food supplies and the implications for food security. *PNAS* 111 4001–4006. 10.1073/pnas.1313490111 24591623 PMC3964121

[B32] Kolde R. (2019). *R Pheatmap: Pretty Heatmaps. R Package Version 1.0.12.*

[B33] Lamb J. F. S. Samac D. A. Barnes D. K. Henjum K. I. (2000). Increased herbage yield in alfalfa associated with selection for fibrous and lateral roots. *Crop Sci.* 40 693–699. 10.2135/cropsci2000.403693x

[B34] Larkin R. P. English J. T. Mihail J. D. (1995). Effects of infection by *Pythium* spp. on root system morphology of alfalfa seedlings. *Phytopathology* 85 430–435. 10.1094/phyto-85-430

[B35] Larkin R. P. English J. T. Mihail J. D. (1996). The relationship of infection by *Pythium* spp. to root system morphology of alfalfa seedlings in the field. *Plant Dis.* 80 281–285. 10.1094/pd-80-0281

[B36] Lerch-Olson E. R. Robertson A. E. (2020). Effect of co-inoculations with *Pythium* and *Fusarium* species on seedling disease development of soybean. *Can. J. Plant Pathol.* 42 408–418. 10.1080/07060661.2019.1668858

[B37] Li K. Guo Q. Zhao L. Chen X. (2009). Study on anastomosis groups and their pathogenicity of *Rhizoctonia solani* isolated from alfalfa in Xinjiang. *Pratacul. Sci.* 26 151–154.

[B38] Li S. Peng X. Wang Y. Hua K. Xing F. Zheng Y. (2019). The effector AGLIP1 in *Rhizoctonia solani* AG1 IA triggers cell death in plants and promotes disease development through inhibiting PAMP-triggered immunity in *Arabidopsis thaliana*. *Front. Microbiol.* 10:2228. 10.3389/fmicb.2019.02228 31611861 PMC6775501

[B39] Liu N. Liu S. Gan Y. Zhang Q. Wang X. Liu S. (2017). Evaluation of mercury resistance and accumulation characteristics in wheat using a modified membership function. *Ecol. Indicators* 78 292–300. 10.1016/j.ecolind.2016.12.025

[B40] Luo D. Tian H. Zhang C. Fang X. (2020). Advances in the research on plant root rot caused by *Rhizoctonia solani*. *China Plant Protection* 40 23–31.

[B41] Michielse C. B. Rep M. (2009). Pathogen profile update: *Fusarium oxysporum*. *Mol. Plant Pathol.* 10 311–324. 19400835 10.1111/j.1364-3703.2009.00538.x PMC6640313

[B42] Morauf C. Steinkellner S. (2015). *Fusarium oxysporum* f. sp. *lycopersici* and compost affect tomato root morphology. *Eur. J. Plant Pathol.* 143 385–398. 10.1007/s10658-015-0691-y

[B43] O’Donnell K. Gueidan C. Sink S. Johnston P. R. Crous P. W. Glenn A. (2009). A two-locus DNA sequence database for typing plant and human pathogens within the *Fusarium oxysporum* species complex. *Fungal Genet. Biol.* 46 936–948. 10.1016/j.fgb.2009.08.006 19715767

[B44] Oladzad A. Zitnick-Anderson K. Jain S. Simons K. Osorno J. M. Mcclean P. E. (2019). Genotypes and genomic regions associated with *Rhizoctonia solani* resistance in common bean. *Front. Plant Sci.* 10:956. 10.3389/fpls.2019.00956 31396253 PMC6667560

[B45] Peters R. D. Grau C. R. (2002). Inoculation with nonpathogenic *Fusarium solani* increases severity of pea root rot caused by *Aphanomyces euteiches*. *Plant Dis.* 86 411–414. 10.1094/pdis.2002.86.4.411 30818716

[B46] Pfender W. F. Hagedorn D. J. (1982). Comparative virulence of *Aphanomyces euteiches* f. sp. *phaseoli* and *Pythium ultimum* on *Phaseolus vulgaris* at naturally occurring inoculum levels. *Phytopathology* 72 1200–1204.

[B47] Rispail N. Rubiales D. (2014). Identification of sources of quantitative resistance to *Fusarium oxysporum* f. sp. *medicaginis* in *Medicago truncatula*. *Plant Dis.* 98 667–673. 10.1094/pdis-03-13-0217-re 30708554

[B48] Rubiales D. Fondevilla S. Chen W. Gentzbittel L. Higgins T. J. V. Castillejo M. A. (2015). Achievements and challenges in legume breeding for pest and disease resistance. *Crit. Rev. Plant Sci.* 34 195–236. 10.1080/07352689.2014.898445

[B49] Schwarz D. Grosch R. (2003). Influence of nutrient solution concentration and a root pathogen (*Pythium aphanidermatum*) on tomato root growth and morphology. *Sci. Hortic.* 97 109–120. 10.1016/s0304-4238(02)00143-7

[B50] Shi S. Nan L. Smith K. F. (2017). The current status, problems, and prospects of alfalfa (*Medicago sativa* L.) breeding in China. *Agronomy* 7:1. 10.3390/agronomy7010001

[B51] Song Q. Liu C. Bachir D. G. Chen L. Hu Y.-G. (2017). Drought resistance of new synthetic hexaploid wheat accessions evaluated by multiple traits and antioxidant enzyme activity. *Field Crops Res.* 210 91–103. 10.1016/j.fcr.2017.05.028

[B52] Sun Q. Liu Q. Na Y. Li F. Tao Y. (2016). The history of the introduced of alfalfa to China in the Han dynasty. *Acta Pratacult. Sin.* 25 240–253.

[B53] Susi H. Barrès B. Vale P. F. Laine A.-L. (2015). Co-infection alters population dynamics of infectious disease. *Nat. Commun.* 6 1–8. 10.1038/ncomms6975 PMC4354079 25569306

[B54] Tollenaere C. Susi H. Laine A.-L. (2016). Evolutionary and epidemiological implications of multiple infection in plants. *Trends Plant Sci.* 21 80–90. 10.1016/j.tplants.2015.10.014 26651920

[B55] Vandemark G. J. Ariss J. J. Hughes T. J. (2010). Real-time PCR suggests that *Aphanomyces euteiches* is associated with reduced amounts of *Phytophthora medicaginis* in alfalfa that is co-inoculated with both pathogens. *J. Phytopathol.* 158 117–124. 10.1111/j.1439-0434.2009.01583.x

[B56] Vu D. Groenewald M. De Vries M. Gehrmann T. Stielow B. Eberhardt U. (2019). Large-scale generation and analysis of filamentous fungal DNA barcodes boosts coverage for kingdom fungi and reveals thresholds for fungal species and higher taxon delimitation. *Stud. Mycol.* 92 135–154. 10.1016/j.simyco.2018.05.001 29955203 PMC6020082

[B57] Wang G. Liu S. Fang Y. Shangguan Z. (2020a). Adaptive changes in root morphological traits of Gramineae and Leguminosae seedlings in the ecological restoration of the semiarid region of northwest China. *Land Degrad. Dev.* 31 1–13. 10.1155/2015/645965

[B58] Wang Z. Tian H. Zhang C. Fang X. Nan Z. (2020b). Occurrence of *Macrophomina phaseolin* a causing root and crown rot on alfalfa (*Medicago sativa*) in China. *Plant Dis.* 104:2521. 10.1094/pdis-03-20-0555-pdn

[B59] Wille L. Messmer M. M. Studer B. Hohmann P. (2019). Insights to plant-microbe interactions provide opportunities to improve resistance breeding against root diseases in grain legumes. *Plant Cell Environ.* 42 20–40. 10.1111/pce.13214 29645277

[B60] Xin B. B. Yuan Q. H. Wang Y. Ma J. Q. (2016). Evaluation on disease resistance of different varieties of alfalfa to *Fusarium oxysporum*. *Chin. J. Grass.* 38 74–80.

[B61] Yan C. Song S. Wang W. Wang C. Li H. Wang F. (2020). Screening diverse soybean genotypes for drought tolerance by membership function value based on multiple traits and drought-tolerant coefficient of yield. *BMC Plant Biol.* 20:321. 10.1186/s12870-020-02519-9 32640999 PMC7346468

[B62] Yang S. Gao M. Xu C. Gao J. Deshpande S. Lin S. (2008). Alfalfa benefits from *Medicago truncatula*: the *RCT1* gene from M. truncatula confers broad-spectrum resistance to anthracnose in alfalfa. *PNAS* 105 12164–12169. 10.1073/pnas.0802518105 18719113 PMC2527883

[B63] You M. P. Barbetti M. J. (2017). Severity of phytophthora root rot and pre-emergence damping-off in subterranean clover influenced by moisture, temperature, nutrition, soil type, cultivar and their interactions. *Plant Pathol.* 66 1162–1181. 10.1111/ppa.12655

[B64] You M. P. Nichols P. Katusiime R. Barbetti M. J. (2020). Novel disease host resistances in the world core collection of *Trifolium subterraneum*. *Plant Dis.* 10.1094/PDIS-1009-1020-1985-RE [Epub ahead of print]. 33107794

