# Supplementary material for: Co-infection by Soil-Borne Fungal Pathogens Alters Disease Responses Among Diverse Alfalfa Varieties
Source: Front Microbiol. 2021 Jul 14;12:664385. doi: 10.3389/fmicb.2021.664385 (PMC8317461; doi:10.3389/fmicb.2021.664385)
Supplement: Supplementary Figure 1 — Disease severity of 80 alfalfa varieties that were single or co-inoculated with Fom and Rs. [file Data_Sheet_1.DOCX]

**Supplementary Material**

**Supplemental Table S1.** Details of alfalfa (*Medicago sativa* subsp. *sativ*a and *M. sativa* subsp. *varia*) varieties used in this study.

| **no.** | **Type*^a^*** | **Variety** | **Origin** | **Year*^b^*** | **Agronomic attribute** | **Suitable area*^c^*** | **Seed source*^d^*** |
| --- | --- | --- | --- | --- | --- | --- | --- |
| *Medicago sativa* subsp. *sativ*a | | |  |  |  |  |  |
| Msv2 | BV | Gannong 3 | China | 1996 | Drought and cold tolerance, high yield | Northwest | GAU |
| Msv4 | BV | Gannong 4 | China | 2005 | High yield | Northwest | GAU |
| Msv5 | BV | Gannong 5 | China | 2009 | Insect resistance, high yield | Northwest | GAU |
| Msv6 | BV | Gannong 6 | China | 2009 | Drought and cold tolerance, high yield | Northwest | GAU |
| Msv7 | BV | Gannong 7 | China | 2013 | High yield, good quality | The North temperate | GAU |
| Msv8 | BV | Gannong 9 | China | 2017 | Thrip resistance, high yield | The North temperate | GAU |
| Msv9 | BV | Zhonglan 1 | China | 1989 | Drought tolerance, downy mildew resistance, high yield | The North | LIHPS |
| Msv10 | BV | Zhonglan 2 | China | 2017 | High yield, good quality | Loess Plateau | LIHPS |
| Msv11 | BV | Dongmu 1 | China | 2010 | Cold tolerance, high yield, good quality | Northeast | NNU |
| Msv12 | BV | Dongmu 3 | China | New | Cold tolerance, high yield, good quality | Northeast | NNU |
| Msv13 | BV | Longmu 803 | China | 1992 | Cold tolerance, high yield | Northeast | HAHRI |
| Msv14 | BV | Longmu 808 | China | 2002 | Drought and saline-alkali tolerance, high yield, good quality | Northeast | HAHRI |
| Msv15 | BV | Longmu 809 | China | 2019 | Cold tolerance | Northeast, North China | HAHRI |
| Msv16 | BV | Gongnong 1 | China | 1987 | Cold tolerance, high yield | Northeast | JAAS |
| Msv19 | BV | Zhongmu 1 | China | 1997 | Cold tolerance, disease resistance, high yield | Huang-Huai-Hai | CAAS |
| Msv20 | BV | Zhongmu 3 | China | 2006 | Salinity tolerance | Huang-Huai-Hai | IAS-CAAS |
| Msv21 | BV | Zhongmu 1 | China | 1998 | Cold and salinity tolerance | Huang-Huai-Hai | IAS-CAAS |
| Msv22 | BV | Zhongmu 2 | China | 2003 | Salinity tolerance, high yield | Huang-Huai-Hai | IAS-CAAS |
| Msv23 | BV | Zhongmu 5 | China | 2014 | Salinity tolerance, high yield | Huang-Huai-Hai | IAS-CAAS |
| Msv24 | BV | Zhongmu 8 | China | 2017 | High yield | Huang-Huai-Hai | IAS-CAAS |
| Msv25 | BV | Zhongmu 9 | China | 2019 | Salinity tolerance, high yield | Huang-Huai-Hai | IAS-CAAS |
| Msv30 | BV | Wocao no 3 | China | New | — | North China | LZU |
| Msv31 | BV | Hangmu 1 | China | 2014 | High yield, good quality | Loess Plateau | LIHPS |
| Msv32 | BV | Huaiyang 1 | China | New | — | The South | LZU |
| Msv26 | LV | Longdong | China | 1991 | Drought and cold tolerance, high yield | Loess Plateau | GAU |
| Msv27 | LV | Longzhong | China | 1991 | Drought and cold tolerance, high yield | Northwest | GAU |
| Msv28 | LV | Wudi | China | 1993 | Saline-alkali tolerance, high yield | East China | CAAS |
| Msv29 | LV | Baoding | China | 2002 | Saline-alkali tolerance, high yield | North China | CAAS |
| Msv34 | IV | Hunter field | Austria | — | — | — | NPGS-USDA |
| Msv35 | IV | Trifecta | Austria | — | — | — | NPGS-USDA |
| Msv39 | IV | Hunter river | Australia | — | — | — | NPGS-USDA |
| Msv36 | IV | Concept | Canada | 2017 | Disease resistance, high yield, good quality | North China, Northwest | GWH |
| Msv49 | IV | snow leopard | Canada | — | Cold tolerance | Northeast, Northwest | Beijing Green |
| Msv50 | IV | Commom | Canada | — | — | — | Huafeng |
| Msv51 | IV | Vinal | Canada | — | Cold, drought and saline-alkali tolerance | North Shaanxi, Gansu, North Ningxia, Inner Mongolia | Huafeng |
| Msv52 | IV | Jieli | Canada | — | Humid tolerance, strong regrowth ability | The South | Huafeng |
| Msv53 | IV | SK3010 | Canada | — | Cold tolerance, high yield | Inner Mongolia | Clover |
| Msv54 | IV | MF4020 | Canada | — | Drought tolerance, high yield | Yellow River region, Southwest | Clover |
| Msv55 | IV | SR4030 | Canada | — | Cold tolerance, high yield | North China, Central China | Clover |
| Msv56 | IV | Algongum | Canada | — | Drought and cold tolerance, brown spot and verticillium wilt resistance | North China, Northwest | Clover |
| Msv57 | IV | Victoria | Canada | 2004 | Phytophthora and root rot resistance, high yield | Qinba Mountains, Sichuan Basin | Clover |
| Msv59 | IV | Gloden empress | Canada | — | Drought tolerance, wide adaptability | North China, Northeast, Northwest | Clover |
| Msv60 | IV | AC Caribou | Canada | — | Cold tolerance, drought tolerance, high yield | Northeast | Clover |
| Msv71 | IV | Liangmu 2 | Canada | — | Drought and cold tolerance, disease resistance | North China, Northeast, Northwest | Yasheng |
| Msv80 | IV | Aurora | Canada |  | Cold tolerance, durable | Northeast, Inner Mongolia, Gansu | Huafeng |
| Msv37 | IV | Orca | France | — | — | — | NPGS-USDA |
| Msv48 | IV | Sanditi | France | 2002 | Cold tolerance, disease and insect resistance, high yield | North, Northwest, Central, Southwest | Barenbrug |
| Msv38 | IV | Aurora | Guatemala | — | — | — | NPGS-USDA |
| Msv40 | IV | Arc | USA | — | — | — | NPGS-USDA |
| Msv41 | IV | Saranac AR | USA | — | — | — | NPGS-USDA |
| Msv42 | IV | UC-1465 | USA | — | — | — | NPGS-USDA |
| Msv43 | IV | UC-1887 | USA | — | — | — | NPGS-USDA |
| Msv44 | IV | WL232 | USA | 2004 | Cold tolerance, high yield | North China, Northwest , Northeast | Zhongzhong |
| Msv45 | IV | WL316 | USA | — | high yield | — | Zhongzhong |
| Msv46 | IV | Phabulous | USA | 2004 | High yield, good quality | — | Clover |
| Msv47 | IV | Derby | USA | 2004 | Leaf disease resistance, high yield | North China, Northwest | Barenbrug |
| Msv58 | IV | Dry land | USA | — | Drought and cold tolerance, high yield | Northwest | Clover |
| Msv61 | IV | Salt tolerant star | USA | — | Aline-alkali and drought tolerance, high yield | Areas with saline-alkali soil | Clover |
| Msv62 | IV | Magnum-995 | USA | — | Heat and humidity tolerance, high yield, good quality | South China | Clover |
| Msv63 | IV | Magnum-7 | USA | — | High yield, good quality | Northwest | Clover |
| Msv64 | IV | sw5909 | USA | — | — | — | Yasheng |
| Msv65 | IV | sw3826 | USA | — | — | — | Yasheng |
| Msv66 | IV | sw3211 | USA | — | — | — | Yasheng |
| Msv67 | IV | "030" | USA | — | — | — | Yasheng |
| Msv68 | IV | "020" | USA | — | — | — | Yasheng |
| Msv69 | IV | "040" | USA | — | — | — | Yasheng |
| Msv70 | IV | "010" | USA | — | — | — | Yasheng |
| Msv72 | IV | Adrenalin | USA | 2017 | Aline-alkali tolerance, disease resistance, high yield | The North temperate | Beijing Green |
| Msv73 | IV | WL168HQ | USA | 2017 | Cold and drought tolerance, high yield | Jilin, Liaoning, Inner Mongolia | Zhengdao |
| Msv74 | IV | WL319HQ | USA | — | Cold tolerance, high yield, good quality | Northeast, North China, Northwest | Zhengdao |
| Msv75 | IV | WL298HQ | USA | — | Aline-alkali and cold tolerance, high yield, durable | North Shanxi, Hexi Corridor | Zhengdao |
| Msv76 | IV | WL343HQ | USA | 2015 | Cold tolerance, high yield, good quality, durable | South Beijing | Zhengdao |
| Msv77 | IV | WL354HQ | USA | — | Cold tolerance, root rot resistance, high yield, good quality | Northeast, Northwest, North China | Zhengdao |
| Msv78 | IV | WL363HQ | USA | — | Cold tolerance, high yield | Northwest, Southwest | Zhengdao |
| Msv79 | IV | WL366HQ | USA | — | Aline-alkali and cold tolerance, high yield | — | Zhengdao |
| *M. sativa* subsp. *varia* | | |  |  |  |  |  |
| Mvv1 | Bv | Gannong 1 | China | 1991 | Drought and cold tolerance | Northwest | GAU |
| Mvv3 | Bv | Gannong 2 | China | 1996 | Drought and cold tolerance | Northwest | GAU |
| Mvv17 | Bv | Gongnong 3 | China | 1990 | Cold tolerance, grazing resistance | Northeast | JAAS |
| Mvv18 | Bv | Gongnong 6 | China | New | Cold tolerance | Northeast | JAAS |
| Mvv33 | Bv | Hulunbeier | China | 2005 | Cold and drought tolerance, disease and insect resistance, high yield | Inner Mongolia | HGRI |

***^a^***Variety type: bred variety (BV), local variety (LV), introduced variety (IV); ***^b^***Year, year released/introduced in China; ***^c^***Suitable area for planting in China;

***^d^***NPGS-USDA, National Plant Germplasm System of U.S. Department of Agriculture; GAU, Gansu Agricultural University; LIHPS, Lanzhou Institute of Husbandry and Pharmaceutical Sciences; NNU, Northeast Normal University; HAHRI, Heilongjiang Animal Husbandry Research Institute; JAAS, Jilin Academy of Agricultural Sciences; IAS-CAAS, Institute of Animal Sciences, Chinese Academy of Agricultural Science; HGRI, Hulunbeier Grassland Research Institute; GWH, Grassland Workstation of Heilongjiang; Zhongzhong, Beijing Zhongzhong Grass Industry Co. Ltd.; Beijing Green, Beijing Green Animal Husbandry S&T Development Co. Ltd; Clover, Beijing Clover Grass Science and Technology Center; Barenbrug, Barenbrug (Tianjin) International Grass Industry Co. Ltd.; Huafeng, Zhengzhou Huafeng Grass Industry Technology Co. Ltd; Yasheng, Yasheng Pastural Grass Group Co. Ltd.; Zhengdao, Beijing Zhengdao Ecological Technology Co. Ltd; LZU, Yangzhou University.

**Supplemental Table S2.** Statistical single effect and interaction for the two-way analysis of variance of variety and pathogen treatment on the disease severity, growth and biomass allocation traits.

| **Trait** | **Variety** | |  | **Pathogen** | |  | **Variety×Pathogen** | |  | **Residual** | **cv%** |
| --- | --- | --- | --- | --- | --- | --- | --- | --- | --- | --- | --- |
|  | **d.f.** | ***p* value** |  | **d.f.** | ***p* value** |  | **d.f.** | ***p* value** |  | **d.f.** |  |
| DIS | 79 | <.001 |  | 2 | <.001 |  | 158 | <.001 |  | 1680 | 17.7 |
| DIR | 79 | <.001 |  | 2 | <.001 |  | 158 | <.001 |  | 1680 | 23.7 |
| PH | 79 | <.001 |  | 3 | <.001 |  | 237 | <.001 |  | 2240 | 35.7 |
| RL | 79 | <.001 |  | 3 | <.001 |  | 237 | <.001 |  | 2240 | 28.7 |
| DWS | 79 | <.001 |  | 3 | <.001 |  | 237 | <.001 |  | 2240 | 33.6 |
| DWR | 79 | <.001 |  | 3 | <.001 |  | 237 | <.001 |  | 2240 | 26.9 |
| ST | 79 | <.001 |  | 3 | <.001 |  | 237 | <.001 |  | 2240 | 31.7 |
| RT | 79 | <.001 |  | 3 | <.001 |  | 237 | <.001 |  | 2240 | 28.7 |

DIS and DIR, disease severity index of plant shoot and root, respectively; PH, plant height; RL, root length; DWS and DWR, dry weight of shoot and root, respectively; ST, shoot biomass ratio; RT, root biomass ratio.

**Supplemental Table S3.** Statistical single effect and interaction for the two-way analysis of variance of variety and pathogen treatment on the pathogen effect sizes of growth and biomass allocation traits.

| **Trait** | **Variety** | |  | **Pathogen** | |  | **Variety×Pathogen** | |  | **Residual** | **cv%** |
| --- | --- | --- | --- | --- | --- | --- | --- | --- | --- | --- | --- |
|  | **d.f.** | ***p* value** |  | **d.f.** | ***p* value** |  | **d.f.** | ***p* value** |  | **d.f.** |  |
| PH | 79 | <.001 |  | 2 | <.001 |  | 158 | <.001 |  | 1680 | 39.8 |
| RL | 79 | <.001 |  | 2 | <.001 |  | 158 | <.001 |  | 1680 | 38.5 |
| DWS | 79 | <.001 |  | 2 | <.001 |  | 158 | <.001 |  | 1680 | 27.5 |
| DWR | 79 | <.001 |  | 2 | <.001 |  | 158 | <.001 |  | 1680 | 17.2 |
| ST | 79 | <.001 |  | 2 | <.001 |  | 158 | <.001 |  | 1680 | 70.0 |
| RT | 79 | <.001 |  | 2 | <.001 |  | 158 | <.001 |  | 1680 | 50.7 |

PH, plant height; RL, root length; DWS and DWR, dry weight of shoot and root, respectively; ST, shoot biomass ratio; RT, root biomass ratio.

**Supplemental Table S4.** Pearson’s correlation matrix for the disease severity index and pathogen effect sizes of growth and biomass allocation traits across 80 alfalfa varieties that were single or co-inoculated with Fom and Rs.

|  | **Trait** | **DIS** | **DIR** | **PH** | **RL** | **DWS** | **DWR** | **ST** | **RT** |
| --- | --- | --- | --- | --- | --- | --- | --- | --- | --- |
| Fom | DIS |  |  |  |  |  |  |  |  |
|  | DIR | 0.89^***^ |  |  |  |  |  |  |  |
|  | PH | 0.72^***^ | 0.68^***^ |  |  |  |  |  |  |
|  | RL | 0.03 | 0.03 | 0.15 |  |  |  |  |  |
|  | DWS | 0.74^***^ | 0.71^***^ | 0.95^***^ | 0.16 |  |  |  |  |
|  | DWR | 0.60^***^ | 0.59^***^ | 0.75^***^ | 0.30^***^ | 0.78^***^ |  |  |  |
|  | ST | 0.47^***^ | 0.45^***^ | 0.67^***^ | -0.11 | 0.69^***^ | 0.13 |  |  |
|  | RT | -0.49^***^ | -0.47^***^ | -0.68^***^ | 0.10 | -0.69^***^ | -0.18 | 0.96^***^ |  |
| Rs | DIS |  |  |  |  |  |  |  |  |
|  | DIR | 0.79^***^ |  |  |  |  |  |  |  |
|  | PH | 0.82^***^ | 0.80^***^ |  |  |  |  |  |  |
|  | RL | 0.79^***^ | 0.87^***^ | 0.81^***^ |  |  |  |  |  |
|  | DWS | 0.84^***^ | 0.66^***^ | 0.92^***^ | 0.66^***^ |  |  |  |  |
|  | DWR | 0.89^***^ | 0.77^***^ | 0.84^***^ | 0.85^***^ | 0.86^***^ |  |  |  |
|  | ST | 0.81^***^ | 0.90^***^ | 0.87^***^ | 0.92^***^ | 0.72^***^ | 0.78^***^ |  |  |
|  | RT | 0.60^***^ | 0.79^***^ | 0.53^***^ | 0.88^***^ | 0.36^***^ | 0.69^***^ | 0.78^***^ |  |
| Fom:Rs | DIS |  |  |  |  |  |  |  |  |
|  | DIR | 0.77^***^ |  |  |  |  |  |  |  |
|  | PH | 0.84^***^ | 0.81^***^ |  |  |  |  |  |  |
|  | RL | 0.77^***^ | 0.79^***^ | 0.81^***^ |  |  |  |  |  |
|  | DWS | 0.80^***^ | 0.67^***^ | 0.89^***^ | 0.69^***^ |  |  |  |  |
|  | DWR | 0.82^***^ | 0.75^***^ | 0.81^***^ | 0.88^***^ | 0.84^***^ |  |  |  |
|  | ST | 0.78^***^ | 0.83^***^ | 0.88^***^ | 0.86^***^ | 0.67^***^ | 0.70^***^ |  |  |
|  | RT | 0.64^***^ | 0.77^***^ | 0.62^***^ | 0.89^***^ | 0.43^***^ | 0.73^***^ | 0.82^***^ |  |

Fom, single inoculation with Fom; Rs, single inoculation with Rs; Fom:Rs, co-inoculation with Fom and Rs. Significant correlations are shown (*, *p* < 0.05; **, *p* < 0.01; ***, *p* < 0.001). DIS and DIR, disease severity index of plant shoot and root respectively; PH, plant height; RL, root length; DWS and DWR, dry weight of shoot and root, respectively; ST, shoot biomass ratio; RT, root biomass ratio.

**Supplemental Figure S1.** Disease severity of 80 alfalfa varieties that were single or co-inoculated with Fom and Rs. (a) Disease severity index of plant shoot (DIS). (b) Disease severity index of root (DIR). Fom, single inoculation with Fom; Rs, single inoculation with Rs; Fom:Rs, co-inoculation with Fom and Rs. Plants of the control treatment for companions showed no diseases and data were not shown. Bars represent standard error (SE) of means (n = 8) from two replicated experiments.

**
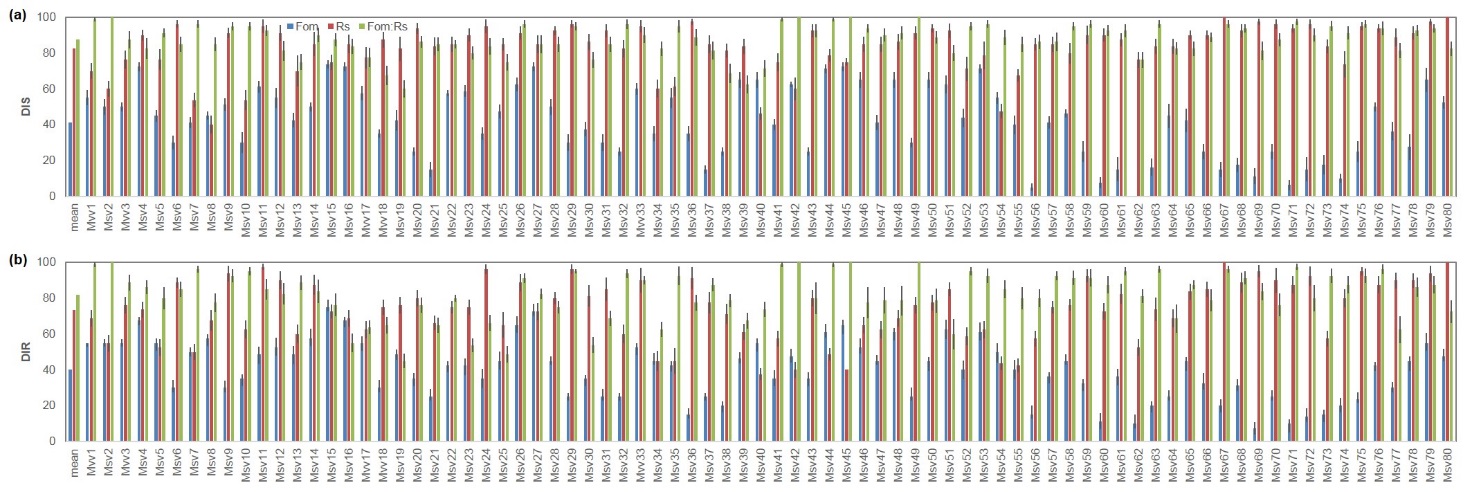
**

**Supplemental Figure S2.** Pathogen effects on plant growth of 80 alfalfa varieties that were single or co-inoculated with Fom and Rs. (a) Pathogen effect sizes of plant height (PH). (b) Pathogen effect sizes of root length (RL). (c) Pathogen effect sizes of dry weight for shoot (DWS). (d) Pathogen effect sizes of dry weight for root (DWR). Fom, single inoculation with Fom; Rs, single inoculation with Rs; Fom:Rs, co-inoculation with Fom and Rs. Bars represent standard error (SE) of means (n = 8) from two replicated experiments.

**
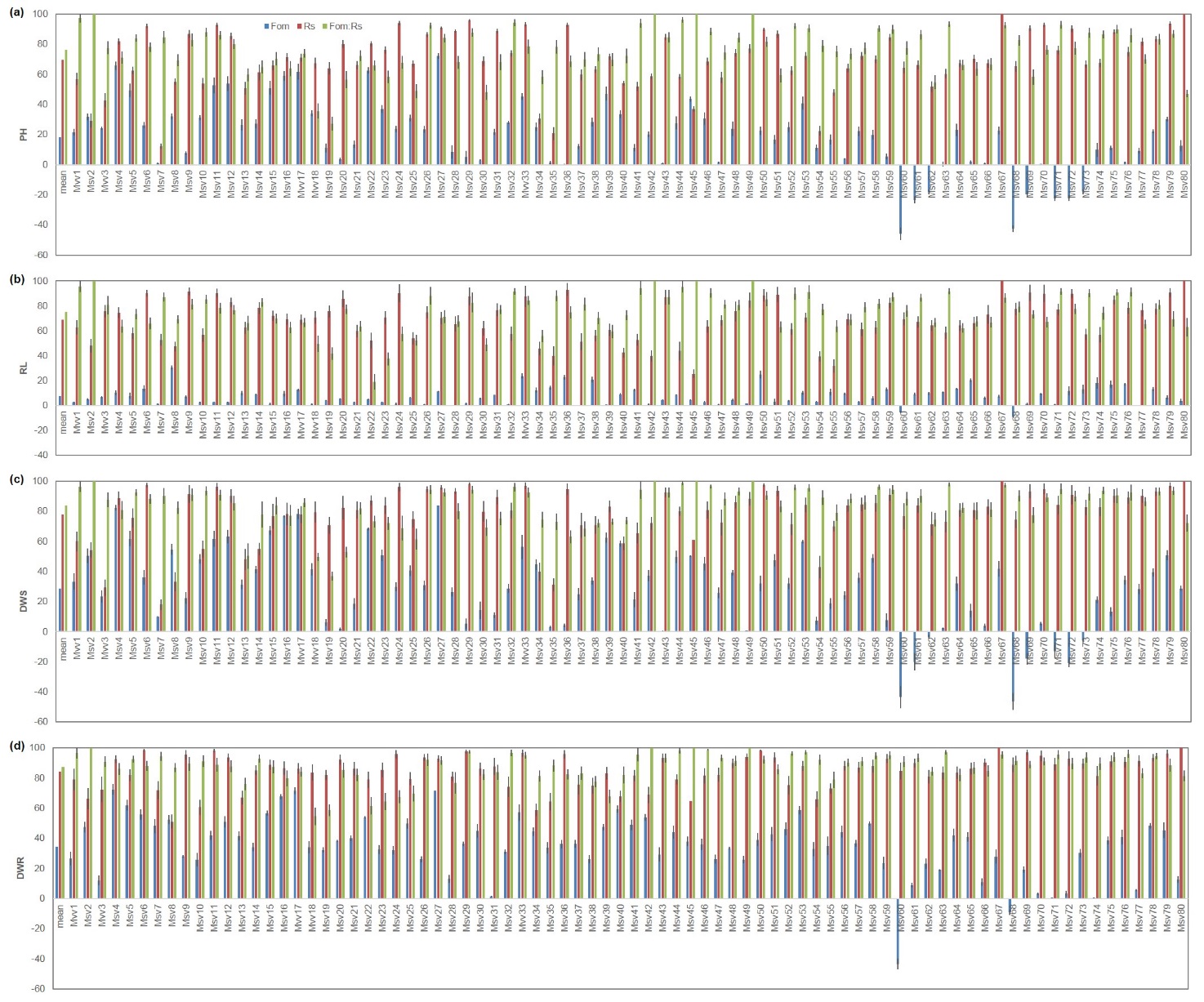
**

**Supplemental Figure S3.** Pathogen effects on biomass allocation of 80 alfalfa varieties that were single or co-inoculated with Fom and Rs. (a) Pathogen effect sizes of shoot biomass ratio (ST). (b) Pathogen effect sizes of root biomass ratio (RT). Fom, single inoculation with Fom; Rs, single inoculation with Rs; Fom:Rs, co-inoculation with Fom and Rs. Bars represent standard error (SE) of means (n = 8) from two replicated experiments.

**
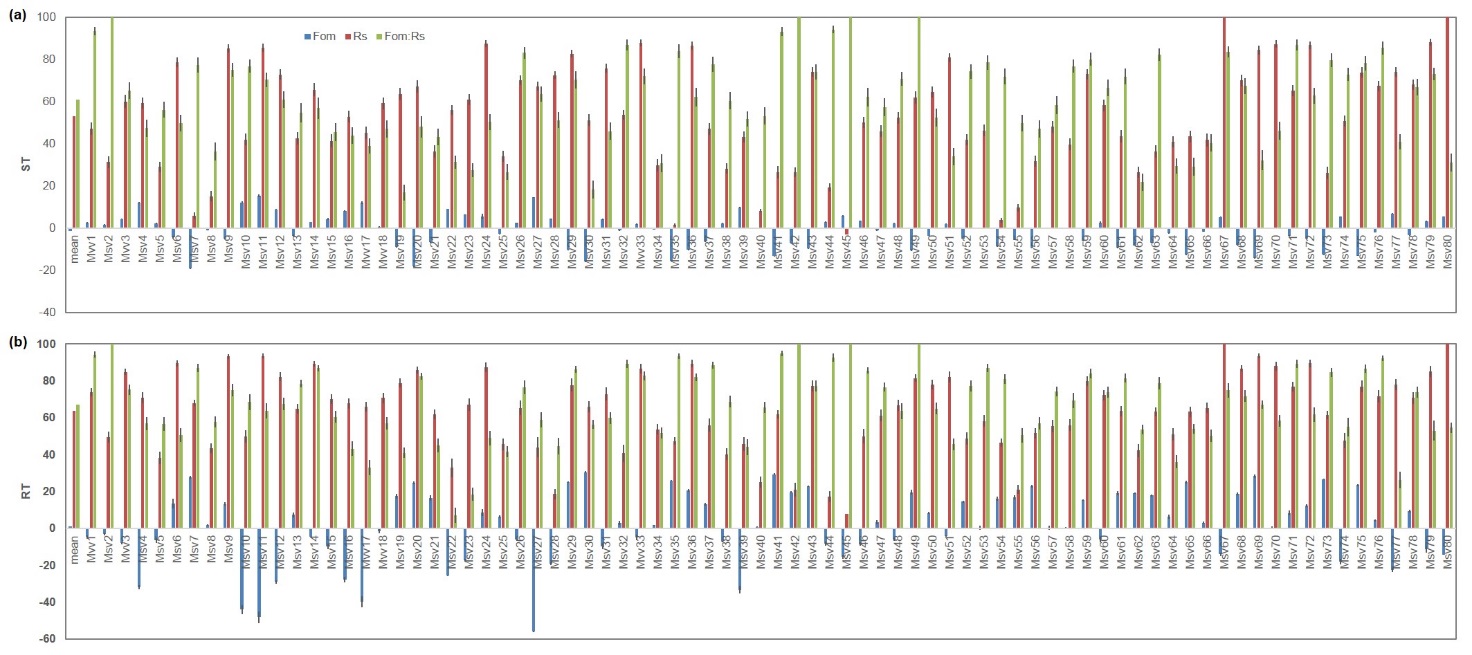
**

**Supplemental Figure S4.** Variations in root morphology among 12 alfalfa varieties that were single or co-inoculated with Fom and Rs. Variations in (a) root diameter (RD), (b) total root length (TRL), (c) root surface area (RSA) and (d) root volume (RV). Fom, single inoculation with Fom; Rs, single inoculation with Rs; Fom:Rs, co-inoculation with Fom and Rs. Boxplots show the medians, 25th and 75th percentiles, with whiskers extending to 1.5 times of the interquartile range, and data presented beyond whiskers represent outliers. Different letters above the bars indicate significant differences (*p* < 0.001) among treatments according to Fisher’s protected least significant difference test at *p =* 0.05.

**
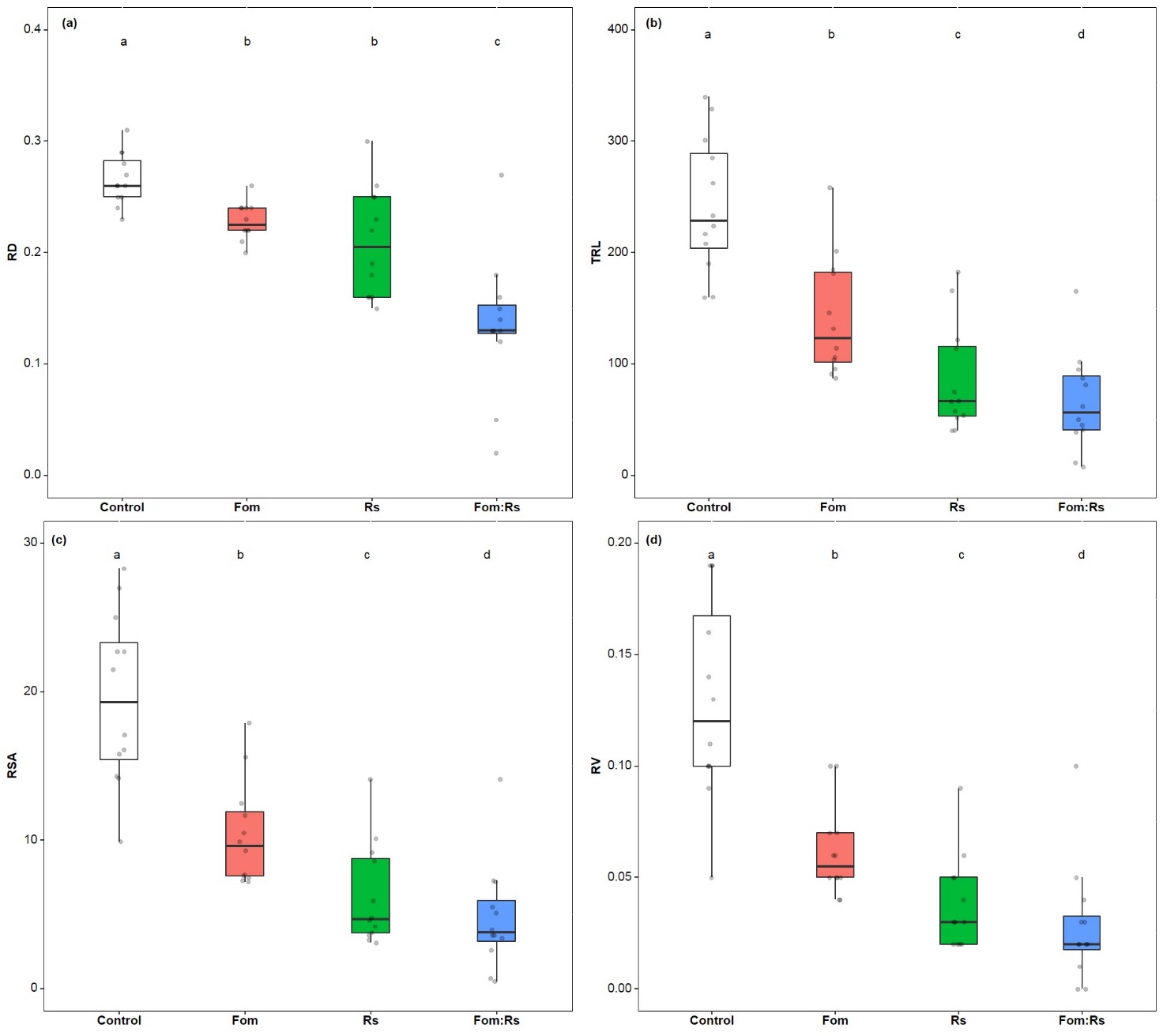
**

**Supplemental Figure S5.** Comprehensive resistance ranking of the 80 alfalfa varieties to Fom and Rs under either single or co-infection based on the membership function value (*D_v_*). (a) Varieties resistance to single infection by Fom (Fom), (b) varieties resistance to single infection by Rs (Rs), and (c) varieties resistance to co-infection by Fom and Rs (Fom:Rs). A larger *Dv* value indicated the resistance of the variety was higher while a smaller value indicated the resistance was lower. The varieties ranked in the top 10 (in orange) were considered as the most resistant varieties under each pathogen treatment.

**
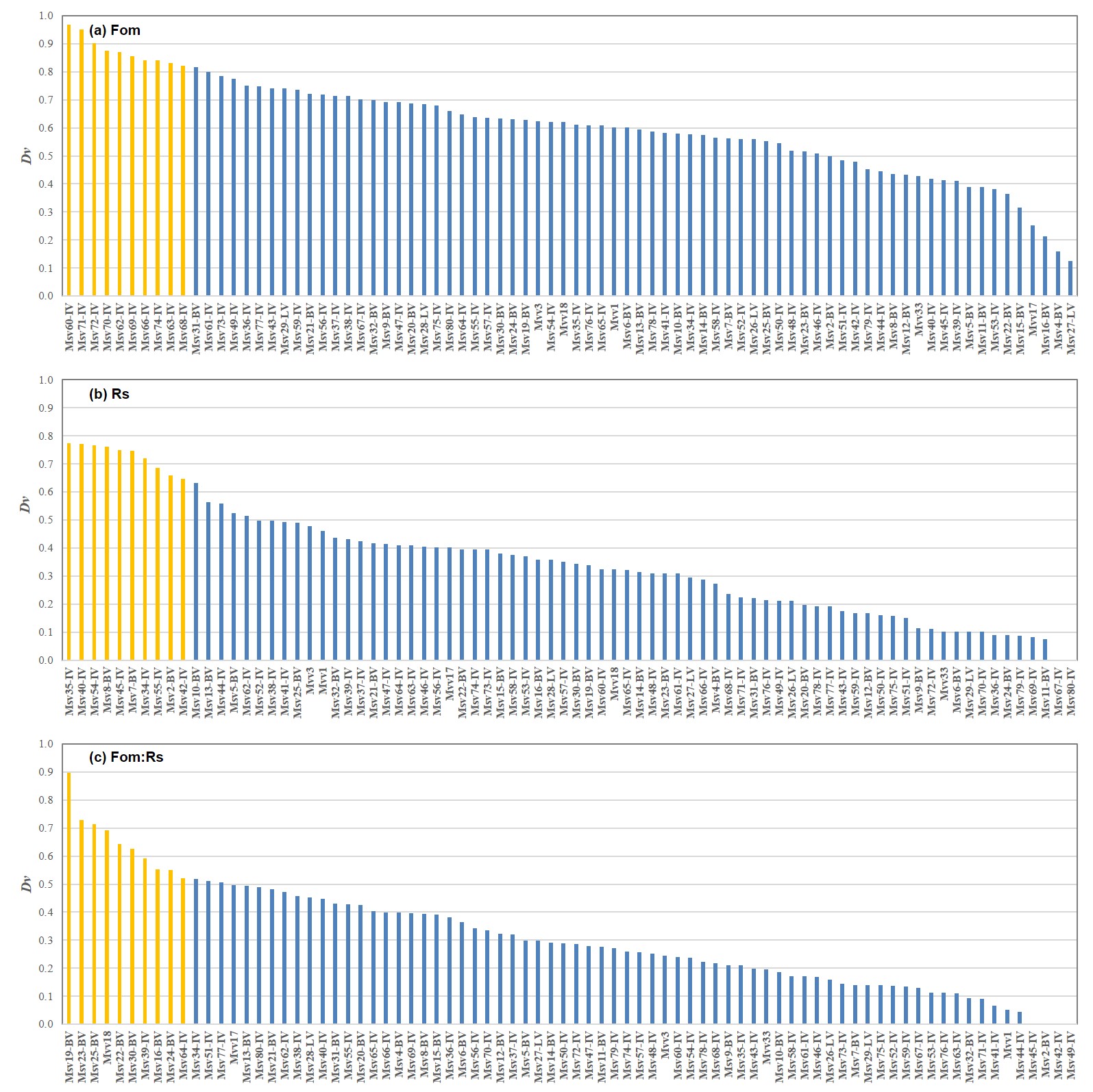
**
